# Investigating the immunological function of alpha-2-glycoprotein 1, zinc-binding in regulating tumor response in the breast cancer microenvironment

**DOI:** 10.1007/s00262-024-03629-1

**Published:** 2024-02-13

**Authors:** Toru Hanamura, Kozue Yokoyama, Shigehisa Kitano, Hiroshi Kagamu, Makiko Yamashita, Mayako Terao, Takuho Okamura, Nobue Kumaki, Katsuto Hozumi, Takayuki Iwamoto, Chikako Honda, Sasagu Kurozumi, Jennifer K. Richer, Naoki Niikura

**Affiliations:** 1 Department of Breast Oncology, Tokai University School of Medicine, 143 Shimokasuya, Isehara-shi, Kanagawa Prefecture 259-1193 Japan; 2 Division of Cancer Immunotherapy Development, Department of Advanced Medical Development, The Cancer Institute Hospital of JFCR, 3-8-31, Ariake, Koto, Tokyo 135-8550 Japan; 3 Division of Respiratory Medicine, Saitama Medical University International Medical Center, 1397-1, Yamane, Hidaka-shi, Saitama Prefecture 350-1298 Japan; 4 Department of Pathology, Tokai University School of Medicine, 143 Shimokasuya, Isehara-shi, Kanagawa Prefecture 259-1193 Japan; 5 Department of Immunology, Tokai University School of Medicine, 143 Shimokasuya, Isehara-shi, Kanagawa Prefecture 259-1193 Japan; 6 Kawasaki Medical School Hospital, Breast and Thyroid Surgery, 577 Matsushima, Kurashiki-shi, Okayama Prefecture 701-0192 Japan; 7 Department of General Surgical Science, Gunma University Graduate School of Medicine, 39-22, Showa-Machi 3-Chome, Maebashi-shi, Gunma Prefecture 371-8511 Japan; 8 Department of Breast Surgery, International University of Health and Welfare, 4-3, Kozunomori, Narita-shi, Chiba Prefecture 286-8686 Japan; 9 Department of Pathology, University of Colorado Anschutz Medical Campus, 12800 East 19th Avenue, Mailstop 8104, Aurora, CO 80045 USA

**Keywords:** Breast cancer, Alpha-2-glycoprotein 1, zinc-binding, Androgen receptor, Tumor immunity, Microenvironment, Macrophage differentiation

## Abstract

**Background:**

Alpha-2-glycoprotein 1, zinc-binding (ZAG), a secreted protein encoded by the *AZGP1* gene, is structurally similar to HLA class I. Despite its presumed immunological function, little is known about its role in tumor immunity. In this study, we thus aimed to determine the relationship between the expression of *AZGP1*/ZAG and the immunological profiles of breast cancer tissues at both the gene and protein level.

**Methods:**

Using a publicly available gene expression dataset from a large-scale breast cancer cohort, we conducted gene set enrichment analysis (GSEA) to screen the biological processes associated with *AZGP1*. We analyzed the correlation between *AZGP1* expression and immune cell composition in breast cancer tissues, estimated using CIBERSORTx. Previously, we evaluated the infiltration of 11 types of immune cells for 45 breast cancer tissues using flow cytometry (FCM). ZAG expression was evaluated by immunohistochemistry on these specimens and analyzed for its relationship with immune cell infiltration. The action of ZAG in M1/M2 polarization models using primary cultures of human peripheral blood mononuclear cells (PBMC)-derived macrophage (Mφ) was analyzed based on the expression of M1/M2 markers (CD86, CD80/CD163, MRC1) and HLA class I/II by FCM.

**Results:**

*AZGP1* expression was negatively correlated with multiple immunological processes and specific immune cell infiltration including Mφ M1 using GSEA and CIBERSORTx. ZAG expression was associated with decreased infiltration of monocytes/macrophages, non-classical monocytes, and myeloid-derived suppressor cells in tumor tissues assessed using FCM. In in vitro analyses, ZAG decreased the expression of CD80, CD163, MRC1, and HLA classes I/II in the M1 polarization model and the expression of CD163 and MRC1 in the M2 polarization model.

**Conclusion:**

ZAG is suggested to be a novel immunoregulatory factor affecting the Mφ phenotype in breast cancer tissues.

**Supplementary Information:**

The online version contains supplementary material available at 10.1007/s00262-024-03629-1.

## Introduction

Worldwide, breast cancer has the highest incidence rate of all cancers among women. Despite the progress of multidisciplinary therapy, it is difficult to cure advanced or recurrent cases [1], thus necessitating the development of innovative treatment strategies. The efficacy of immune checkpoint inhibitors has been demonstrated in multiple cancer types, and tumor immunology has attracted great attention as an innovative therapeutic strategy [2]. Therefore, elucidating the unique immunomodulatory mechanisms underlying the breast cancer microenvironment will provide significant insights toward development of novel therapeutic strategies.

Among all cancer types, androgen receptor (AR) is most highly expressed in breast cancer after prostate cancer (The Human Protein Atlas.org. https://www.proteinatlas.org/ENSG00000169083-AR; accessed on November 07, 2022) [3]. It is expressed in 60–80% of breast cancers with varying extents across the subtypes [4]. AR is a nuclear transcription factor with a diverse range of biological actions, principally in the development and maintenance of the male reproductive system [5]. Accumulating data strongly suggest that AR signaling affects the immune response in various physiological and pathological conditions including allergic disease, autoimmune disease, and cancer [6–8]. Recently, we and other researchers found that AR expression is inversely correlated with immune cell infiltration in breast cancer tissues [9–12]. However, there is limited information regarding its mechanisms. Secreted factors regulated by AR can act as immune-regulation mediators. In a previous study, we identified multiple AR-dependent secreted proteins produced by breast cancer cells [13]. Alpha-2-glycoprotein 1, zinc-binding (ZAG), encoded by the *AZGP1* gene, is one of these proteins and is the second most sensitive protein to AR activity after prostate-specific antigen (so-called PSA). ZAG is a 40-kDa single-chain polypeptide secreted in various body fluids and present in high concentrations in the human seminal plasma and breast cyst fluid [14, 15]. Moreover, ZAG is significantly higher in the serum of patients with breast cancer than in healthy controls [16]. Owing to its structural similarity to HLA class I, ZAG is thought to be involved in immune response, although its detailed function remains unclear [14, 17].

In this study, we focused on ZAG as a candidate protein to regulate AR-dependent immune-regulatory mechanisms in the breast cancer microenvironment. We systematically analyzed the relationship between the expression of *AZGP1*/ZAG and the immunological profiles of breast cancer tissues at both the gene and protein level. Furthermore, we verified the effect of ZAG on Mφ using in vitro models of primary culture of human peripheral blood mononuclear cell (PBMC)-derived Mφ.

## Materials and methods

### Gene expression profile datasets

We utilized two publicly available gene expression profile datasets of a large breast cancer cohort, namely the Molecular Taxonomy of Breast Cancer International Consortium (METABRIC) [18] cohort (*n* = 1904) and the Sweden Cancerome Analysis Network-Breast (SCAN-B) [19] cohort (*n* = 3273), generated using microarray and RNA sequencing, from the cBioPortal (https://www.cbioportal.org/ accessed on February 20, 2019) and Gene Expression Omnibus (https://www.ncbi.nlm.nih.gov/geo/ accessed on June 16, 2019), respectively.

## Gene set enrichment analysis (GSEA)

We performed gene set enrichment analysis (GSEA) using the GSEA software v4.0 (https://www.gsea-msigdb.org/gsea/msigdb/ accessed on November 21, 2019). The log2 transformed gene expression values in the METABRIC dataset were applied to GSEA [20]. Hallmark gene set collections (50 gene sets) representing specific well-defined biological states or processes, obtained from MsigDB v7.1 (https://www.gsea-msigdb.org/gsea/msigdb/ accessed on May 13, 2020), were set as the gene set database. Expression values of *AZGP1* were used as phenotype labels, and Pearson’s correlation was used for ranking genes. Other parameters were set at defaults. We set the thresholds for nominal *p* values and false discovery rate *q*-values at < 0.05 and < 0.25, respectively. The SCAN-B dataset was not used for GSEA because of the incompatible normalization method.

## CIBERSORTx

The non-log-transformed gene expression data from METABRIC and SCAN-B were applied to CIBERSORTx (https://cibersortx.stanford.edu/ accessed on February 25, 2022) [21] with “LM22” [22] set as the signature matrix file. The program was run in the absolute mode with 100 permutations. We applied the B-mode batch correction and set quantile normalization as disabled. Eventually, we estimated the absolute abundance of total immune cells (i.e., absolute scores) and that of 22 functionally defined human immune cell types in bulk tumor tissues. Cases with a CIBERSORTx *p* value < 0.05 were filtered and selected for subsequent analyses.

## Participants

We used data from our previous study of immune cell composition in breast cancer tissues from 45 cases using flow cytometry (FCM) [9, 23]. For the preparation of PBMC-derived Mφ, we recruited healthy women (*n* = 39, median 43 years; range 22–63 years) who did not use steroids, immunosuppressants, hormonal agents, oral contraceptives, or endocrine therapy and had no history of primary immunodeficiency, human immunodeficiency virus infection, cancer, or hematologic malignancies.

## Histological evaluation of hormone receptors, human epidermal growth factor receptor 2, Ki67, tumor immunity-related biomarkers, and ZAG

Histological evaluation of hormone receptors (ER: estrogen receptor; PgR: progesterone receptor; and androgen receptor: AR), human epidermal growth factor receptor 2 (HER-2), Ki67, and tumor immunity-related biomarkers (programmed death-ligand 1; PD-L1, tumor-infiltrating lymphocyte; TILs) of breast cancer tissues have been described previously [9, 23]. Histologically assessed PD-L1 and TIL are indicated with “h” (i.e., hPD-L1, hTIL) to distinguish them from others. The nuclear staining of ER, PgR, Ki67, and AR in carcinoma cells was assessed, and the percentage of immunoreactive cells was determined. We determined the ER, PgR, and HER-2 status according to the relevant American Society of Clinical Oncology/College of American Pathologists guidelines [24, 25]. The Ki67 Labeling Index (Ki67 LI) and AR status were categorized into high and low, with cutoff values set at 20% and 60%, respectively [9]. According to the International TILs Working Group guidelines [26], hTILs in the stromal tissue sections were evaluated and categorized into low, intermediate, and high. Tumors with ≥ 1% immune cells displaying cytoplasmic and/or membrane PD-L1 staining were determined to be hPD-L1 positive [27]. ZAG expressions were evaluated using immunohistochemistry and scored using the semiquantitative H-score method [28] to calculate the sum of the percentage and intensity of positively stained invasive tumor cells. Representative examples of ZAG immunostaining are shown in Supplementary Figure S1. ZAG status (i.e., ZAG high, *n* = 22; ZAG low, *n* = 23) was divided by the median value of the H-score. Details of the antibodies used for immunohistochemistry are summarized in Supplementary Table S1.

## TIL preparation from tumor tissues

Details of TIL preparation from breast cancer tissues have been described previously [23]. Briefly, fresh breast cancer tissues were mechanically dissociated and filtered using a cell strainer to obtain a cell suspension. Subsequently, mononuclear cell components were separated using density gradient centrifugation with a Ficoll-Paque PLUS (Cytiva Inc., Tokyo, Japan). They were suspended in a CELLBANKER I (Takara Bio Inc. Shiga, Japan) and stored in liquid nitrogen until the FCM analysis.

## Monocyte isolation

PBMCs were separated from the whole blood sample of healthy women by density gradient centrifugation using Histopaque^®^ 1077 (Sigma-Aldrich, St. Louis, MO, USA). Red blood cells were hemolyzed with a red blood cell lysing buffer (Sigma-Aldrich, St. Louis, MO, USA) for 10 min at 37 °C. The cell suspension was treated with a FcR blocking reagent (Miltenyi Biotech, Bergisch, Gladbach, Germany) and Cluster of Differentiation (CD) 14 MicroBeads (Miltenyi Biotech, Bergisch, Gladbach, Germany) for 15 min at 4 °C. The cells were washed, and the CD14 + monocytes were isolated using an autoMACS^®^ Pro Separator (Miltenyi Biotech K.K., Tokyo, Japan).

## Construction of M1/M2 polarization models using PBMC-derived Mφ and THP-1 cells

Monocytes isolated from PBMCs were suspended in a basal medium consisting of RPMI 1640 media (Gibco Brl, Grand Island, NY, USA), supplemented with heat-inactivated 10% fetal bovine serum (BioWest, Nuaillé, France) and 1% penicillin–streptomycin (Sigma-Aldrich, St. Louis, MO, USA), counted and seeded into Upcell^®^ Multi 24 well plate (CellSeed Inc, Tokyo, Japan) in 2E5 to 5E5 cells/well. To obtain Mφ [29], we incubated the monocytes for 4 days in a basal medium supplemented with 10 ng/mL granulocyte macrophage colony-stimulating factor (GM-CSF) (Peprotech, Rocky Hill, NJ, USA) at 37 °C and 5% CO_2_. Subsequently, we replaced the medium with a basal medium comprising each of the following supplement: M1 polarization model—with 10 ng/mL interferon gamma (Peprotech, Rocky Hill, NJ, USA) + 10 pg/mL lipopolysaccharides from *Escherichia coli* (LPS, Sigma-Aldrich, St. Louis, MO, USA) [30]; M2 polarization model—with 20 ng/mL interleukin 4 (Peprotech, Rocky Hill, NJ, USA) [31]; and non-polarization model—no supplement. In all polarization models, the indicated concentrations of recombinant human-ZAG (R&D Systems, Minneapolis, MN, USA) or vehicle control were added; after 2 days of incubation, the cells were harvested and subjected to an FCM analysis (Supplementary Figure S2a–c). To harvest the cells, the medium was replaced by ice-cold phosphate buffered saline to the Upcell^®^ Multi 24-well plate. The plates were maintained at 25 °C for 30 min to promote cell detachment, and the cells were collected by pipetting. Furthermore, we examined human monocytic THP-1 [32] cells for a similar experiment with minor modifications. Briefly, THP-1 cells were supplied by Dr. A. Kotani, cultured in a basal medium and culture conditions similar to that of monocytes isolated from PBMC. We used 10 ng/ml Phorbol-12-myristate-13-acetate (PMA) (Adipogen Corp. San Diego, CA, USA) for Mφ differentiation, instead of GM-CSF, and performed each experiment in triplicate (Supplementary Figure S3a–c).

## FCM analysis

FCM data from breast cancer tissue samples were obtained from our previous study [23] and used for this ad hoc analysis. According to the staining profile of the FCM-evaluated surface antigen, the cells were classified as follows: leukocytes, total T cells, CD4 + T cells (CD4 + T), CD8 + T cells (CD8 + T), B cells (B), monocytes/macrophages (Mo/Mφ), non-classical monocytes (CD16 + Mo), myeloid-derived suppressor cells (MDSCs), dendritic cells (DCs), myeloid DCs, natural killer (NK) cells, minor NK cells, and natural killer T cells. The density of each immune cell fraction was determined as the count of cells per weight of the tumor tissue (count/g) [23]. Furthermore, we assessed the percentage of PD-L1 and CD86 positive cells in each immune cell fraction. The gating strategy for PD-L1 or CD86 positivity is described in Supplementary Figure S4a-d. We examined the action of ZAG in M1, M2, and non-polarization models based on the expression of surface antigens, such as M1/M2 polarization markers (CD86, CD80/CD163, and MRC1) [33] and HLA class I/II, evaluated using FCM (*n* = 15). The cell suspension was supplemented with FcR blocking reagent and mixed with an antibody cocktail. The cells reacted at 4 °C for 30 min and washed. Stained samples were detected using BD LSR Fortessa (BD Biosciences, Franklin Lakes, NJ, USA) and analyzed using the FlowJo software v10.8.1 (BD Biosciences). The gating strategy for single-cell detection and representative histograms of fluorescence intensity derived from each surface antigens are described in Supplementary Figure S5a, b. Antibodies used in these experiments are summarized in Supplementary Table S2.

## Statistical analyses

We used the GraphPad Prism ver. 9.1.0 software for statistical analyses and graph preparation. All data were assessed using the D'Agostino–Pearson normality test; parametric or nonparametric tests were used depending on the data distribution. We performed correlation analyses between the groups using Spearman’s rank correlation coefficient. |*r*-value|> 0.3, and a significant *p* value was defined as a positive or negative correlation [34]. The Fisher’s exact test was performed to compare categorical variables between the groups. Continuous variables between the two unpaired groups were compared using the unpaired t test or the Mann–Whitney U test. For multiple comparisons, we performed the Kruskal–Wallis test and Dunn's multiple comparisons test. Continuous variables between two paired groups were compared using the paired* t* test or the Wilcoxon-test. A *p* value < 0.05 was defined as statistically significant. In our previous study, FCM data from patients with breast cancer contained outliers [23]. Here, all analyses were performed without omitting outliers; nonetheless, we identified the outliers using robust regression and outlier removal method, excluded them, and performed all statistical analyses to ensure the reliability of our analyses.

## Results

### Biological process and immunological profile associated with the gene expression levels of AZGP1 in breast cancer tissues

**Fig. 1:**
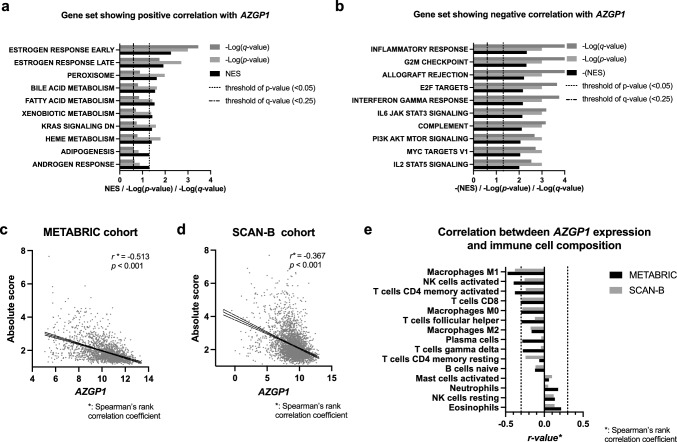
Biological process and immunological profile associated with *AZGP1* gene expression levels in breast cancer tissues **a**, **b** GSEA results from the METABRIC datasets. Biological processes positively or negatively correlated with *AZGP1* gene expression are depicted in a descending order of the absolute value of the normalized enrichment score (NSE), with absolute values of the log-transformed nominal *p* values and FDR *q*-values. Thresholds of the nominal *p* value and FDR *q*-value are set to < 0.05 and < 0.25, respectively, and the boundaries are depicted in the graph by dashed lines. **c**, **d** Scatterplots displaying a correlation between *AZGP1* expression levels and the absolute score estimated by CIBERSORTx. Solid and dashed lines indicate the regression line and 95% confidence band, respectively. **e** Graphs displaying the correlation coefficient (*r*-value) between *AZGP1* expression and the absolute amount of various immune cell fractions estimated by CIBERSORTx. Immune cell fraction data displaying consistently significant in the METABRIC and SCAN-B datasets have been depicted in an ascending order of the *r*-value. Abbreviations: FDR, false discovery rate; GSEA, Gene Set Enrichment Analysis; METABRIC, Molecular Taxonomy of Breast Cancer International Consortium; NSE, normalized enrichment score; and SCAN-B, Sweden Cancerome Analysis Network-Breast

Using the METABRIC dataset, we performed GSEA to screen the biological processes associated with *AZGP1* expression in breast cancer tissues. *AZGP1* expression was positively correlated with a gene set termed ESTROGEN RESPONSE EARY, ESTROGEN RESPONSE LATE, PEROXISOME, BILE ACID METABOLISM, FATTY ACID METABOLISM, XENOBIOTIC METABOLISM, KRAS SIGNAL DN, and HEME METABOLISM (Fig. 1a); it was negatively correlated with INFLAMMATORY RESPONSE, G2M CHECK POINT, ALLOGRAFT REJECTION, E2F TARGETS, INTERFERON GAMMA RESPONSE, IL6 JAK STAT3 SIGNALING, COMPLEMENT, PI3K AKT MTOR SIGNALING, MYC TARGETS V1, and IL2 STAT5 SIGNALING (Fig. 1b). Using the METABRIC and SCAN-B gene expression dataset, we analyzed the correlation between *AZGP1* expression and the immune cell composition in breast cancer tissues estimated with the CIBERSORTx. *AZGP1* expression was negatively correlated with the absolute score demonstrating the absolute abundance of total immune cell infiltration in both the METABRIC and SCAN-B cohort (*r* < − 0.3 and *p* < 0.05) (Fig. 1c, d). *AZGP1* expression levels were inversely correlated with Mφ M1, NK cells activated, CD4 + T (memory activated), and CD8 + T in at least one dataset (*r* < − 3 and *p* < 0.05) (Fig. 1e). Therefore, *AGZP1* expression was associated with the immune-suppressive phenotype, decreased total immune content, and decreased infiltration of specific immune cell fractions in breast cancer tissues.

## Correlation of the clinicopathological factors and histologically assessed tumor immunity-related biomarkers with ZAG expression

**Fig. 2:**
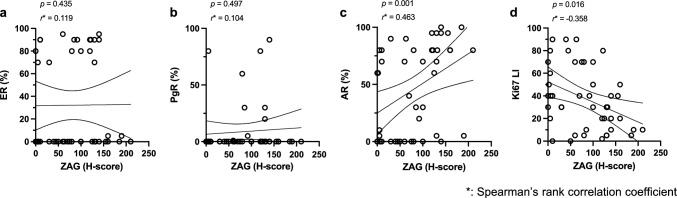
Correlation between ZAG H-score with the percentage of immunoreactive cells for hormone receptors and Ki67 **a–d** Scatter plots depict the correlation between the ZAG H-score with the percentage of immunoreactive cells for the hormone receptors and Ki67. Solid and dashed lines indicate the regression line and 95% confidence band, respectively. The relationship between these two values is analyzed by Spearman’s correlation. |r-value|> 0.3, and a significant *p* value is defined as a positive or negative correlation. Abbreviations: ZAG: Alpha-2-glycoprotein 1, zinc-binding

The high ZAG expression was significantly associated with a lower histological grade and HER2 positivity (Table 1). Despite being insignificant, the AR status and TIL demonstrated a marginal association with the ZAG status, displaying a tendency that ZAG high cases were AR high and TIL low (0.05 < *p* < 0.1). The ZAG H-score demonstrated a positive (*r* > 3 and *p* < 0.05) and negative (*r* < − 3 and *p* < 0.05) correlation with AR and Ki 67 expressions, respectively, but not with ER and PgR (Fig. 2a–d).

**Table 1:** Clinicopathological characteristics by ZAG status

	ZAG high (N = 22)	ZAG low (N = 23)	*p* value*		ZAG high (N = 22)	ZAG low (N = 23)	*p* value*
Menopausal status			0.213	Histological Grade			0.004
Unknown	1	0		Unknown	3	2	
Post	16 (76.2%)	13 (56.5%)		Grade 3	1 (5.3%)	10 (47.6%)	
Pre	5 (23.8%)	10 (43.5%)		Grade 1 or 2	18 (94.7%)	11 (52.4%)	
Neo-adjuvant therapy			1.000	ER status			0.136
absent	18 (81.8%)	19 (82.6%)		Positive (≥ 1%)	12 (54.5%)	7 (30.4%)	
present	4 (18.2%)	4 (17.4%)		Negative (< 1%)	10 (45.5%)	16 (69.6%)	
Histological Type			0.498	PgR status			0.284
IDC	18 (81.8%)	21 (91.3%)		Positive (≥ 1%)	6 (27.3%)	3 (13.0%)	
ILC	1 (4.5%)	0 (0%)		Negative (< 1%)	16 (72.7%)	20 (87.0%)	
Special	3 (13.6%)	2 (8.7%)		AR status			0.075
Invasive tumor size			0.698	High (≥ 60%)	15 (68.2%)	9 (39.1%)	
Unknown	0	1		Low (< 60%)	7 (31.8%)	14 (60.9%)	
≥ 20 mm	17 (77.3%)	19 (86.4%)		HER2 status			0.049
< 20 mm	5 (22.7%)	3 (13.6%)		Unknown	0	1	
Lymph node metastasis			0.227	Positive	7 (31.8%)	1 (4.5%)	
Unknown	0	1		Negative	15 (68.2%)	21 (95.5%)	
Positive	13 (59.1%)	8 (36.4%)		Ki67 LI			0.189
Negative	9 (40.9%)	14 (63.6%)		≥ 20%	14 (63.6%)	19 (82.6%)	
Lymphatic invasion			0.223	< 20%	8 (36.4%)	4 (17.4%)	
Unknown	0	1		TIL			0.080
Negative	7 (31.8%)	12 (54.5%)		Unknown	1	0	
Positive	15 (68.2%)	10 (45.5%)		Low	14 (66.7%)	9 (39.1%)	
Vascular invasion			1.000	Intermediate/High	7 (33.3%)	14 (60.9%)	
Unknown	0	1		PD-L1			0.127
Negative	19 (86.4%)	19 (86.4%)		Unknown	1	0	
Positive	3 (13.6%)	3 (13.6%)		Positive	9 (42.9%)	16 (69.6%)	
				Negative	12 (57.1%)	7 (30.4%)	

^*^Fisher's exact test

## Association of the ZAG status with leukocyte density and tumor-infiltrating immune cells in breast cancer tissues

**Fig. 3:**
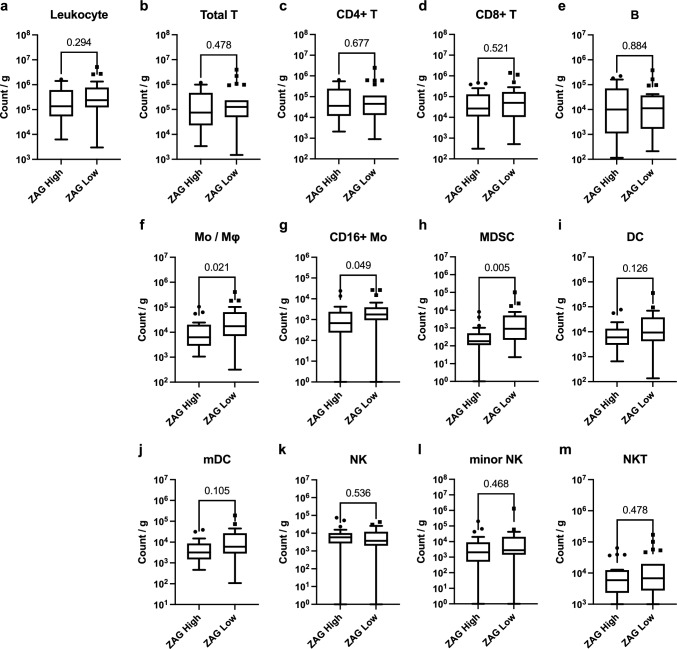
Association between the ZAG status and subsets of tumor-infiltrating immune cells. ZAG status (i.e., ZAG high; *n* = 22, and ZAG low; *n* = 23) were divided by the median value of the H-score. **a** Total leukocyte density (count/g) in breast cancer tissues according to the ZAG status. **b**–**m** Count of each immune cell fraction per unit weight of the tissue (count/g) according to the ZAG status. Unpaired t test or Mann–Whitney U test was conducted depending on the data distribution. The statistical test method, statistics values, and recalculated values, excluding outliers, are summarized in the supplementary tables S3 and S4). Actual *p* values are denoted at the top. Abbreviations: ZAG: Alpha-2-glycoprotein 1, zinc-binding

ZAG expression was associated with the decreased infiltration of Mo/Mφ, CD16 + Mo, and MDSC in breast cancer tissues, but not with the other lineage (Fig. 3a–m). ZAG expression was associated with decreased PD-L1-positive cells in CD4 + T and decreased CD86-positive cells in CD4 + T, CD8 + T, and Mo/Mφ; however, it was not significantly associated with the other lineage (Supplementary Figure S6a–k, S7a–k). The statistical test methods, statistical values, and recalculated values, excluding outliers, are described in Supplementary Table S3–8.

## Action of ZAG in M1/M2 polarization models using PBMC-derived Mφ and THP-1

**Fig. 4:**
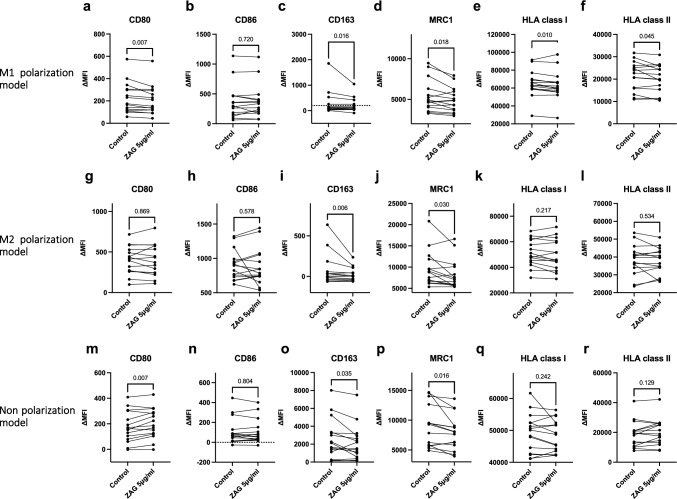
Action of ZAG in PBMC-derived Mφ in vitro. The expression levels of the indicated surface antigens are denoted as ΔMFI by subtracting the MFI of the isotype control from their MFI. The graph depicts the expressions of the indicated surface antigens, with and without ZAG 5 μg/mL in the M1 polarized **a**–**f**, M2 polarized **g**–**l**, and non-polarized condition **m**–**r** (*n* = 15; for each experiment). Paired t test or the Wilcoxon-test was conducted depending on the data distribution. Actual *p* values are denoted at the top. Abbreviations: PBMC, human peripheral blood mononuclear cells; MFI, mean fluorescence intensity; ZAG: alpha-2-glycoprotein 1, zinc-binding; and Mφ, macrophage

The results of the above analysis showing the significant association between Mφ and *AZGP1*/ZAG expression in breast cancer tissue suggest Mφ as a target of ZAG and demonstrates a potential effect on M1 polarization. Accordingly, we analyzed the action of recombinant ZAG in PBMC-derived Mφ grown in the M1/M2 polarized or non-polarized condition (n = 15 for each condition; Supplementary Figure S2a–c). Recombinant ZAG (5 μg/mL) decreased the expression of CD80, CD163, MRC1, and HLA classes I and II in the M1 polarization model (Fig. 4a, c–f) and that of CD163 and MRC1 in the M2 polarization model (Fig. 4i,j). In the non-polarized model, ZAG (5 μg/mL) increased the expression of CD80 and decreased the expression of CD163 and MRC1. Similar analyses using Mφ-like cells derived from THP-1 (n = 3 for each condition; Supplementary Figure S3a–c) demonstrated that recombinant ZAG (5 μg/mL) decreased CD163 and HLA class I expression in the non-polarization model (Supplementary Figure S8o and q). Other surface antigens did not demonstrate significant changes in Dunn's multiple comparison test (Supplementary Figures S8a–r).

## Discussion

This study aimed to assess the role of ZAG protein in the AR-dependent immune-regulatory mechanisms in the breast cancer microenvironment. In silico analyses using public gene expression datasets and analysis of our in-house FCM datasets demonstrated that *AZGP1*/ZAG is associated with immunosuppressive phenotype and reduces the infiltration of specific immune cell subsets, particularly Mφ, into breast cancer tissues. The in vitro analysis using PBMC-derived Mφ indicates that ZAG affects Mφ phenotypic change or function.

In a previous study, we identified seven AR-responsive genes that encode secreted factors in breast cancer [13]. GSEA demonstrated that four of these seven genes were significantly associated with immune-related processes (positive association: *CFH* and *FASN*; negative association: *AZGP1* and *PIP*) (Supplementary Figure S9). Of these, ZAG suppresses inflammatory cytokines and exhibits anti-inflammatory effects in animal models of non-alcoholic fatty liver, epilepsy, and atopic dermatitis [35–37]. However, the function of ZAG in tumor immunity and its specific mechanism are still unknown.

ZAG is a well-known AR regulated protein [14]; however, its significance in breast cancer has not been reported except in association with a lower histologic grade [14]. Analyses of our in-house dataset further supported these existing data and generated novel findings, such as the association of ZAG with lower Ki67 and HER2 positivity (Table 1, Fig. 2). The negative association between ZAG with Ki67 was consistent with the GSEA results, suggesting a negative correlation between *AZGP1* expression and gene sets, such as "G2M CHECK POINT" and "E2F TARGETS" (Fig. 1b). Several reports have shown fewer TILs in hormone receptor-positive HER2-negative subtype compared with that in HER2-positive subtype [38, 39]. Therefore, in this study, it was important to exclude the influence of HER2 status on the tumor immune microenvironment using subgroup or multivariate analysis. However, as mentioned later, the number of cases was small and such an analysis was not possible in the present study; nonetheless, this should be considered in future studies.

This novel study demonstrated the relationship between the immunological profile of the breast cancer microenvironment and *AZGP1*/ZAG expression, with results consistent with the findings of previous reports, suggesting the immunosuppressive functions of ZAG in animal models of various diseases [35–37]. ZAG expression was associated with a lower PD-L1 positive ratio in CD4 + T; however, in our previous studies, PD-L1 expression was originally low in CD4 + T cells [40] and was expressed principally in the myeloid lineage [23]. CD80 and CD86 are predominantly expressed on antigen-presenting cells and bind to CD28 or CLTA-4 on T cells to provide co-stimulatory signals for T-cell activation or inactivation [41]. In addition to the inverse correlation between Mφ and *AZGP1*/ZAG expression in breast cancer tissues (Fig. 1e, 3f), ZAG expression was associated with decreased CD86-positive cells in Mo/Mφ (Supplementary Figure S7d), thus suggesting that ZAG is involved in Mφ differentiation or its function. These molecules are not mere markers; they play a major role in regulating the immune system. For example, both CD80 and CD86 are used as M1 polarization markers [29, 33], and their immunological functions have been discussed. CD163, an M2 marker, has been associated with anti-inflammatory functions [42]. MRC1, an M2 marker [43], is associated with antigen recognition and processing for antigen presentation on HLA class I molecules (cross-presentation); it also has an active role in the induction of T-cell tolerance [44]. Activation of anti-tumor T cells requires the recognition of cancer antigen presented on HLA class I molecules on the tumor; the loss of HLA class I on the tumor leads to the malfunction of recognition by the CD8 + T cells [45, 46]. Furthermore, HLA class II molecules are used as M1 markers [33] and are involved in antigen presentation to CD4 + T helper cells.

In addition to relatively high expression in the mammary gland, *AZGP1* is highly expressed in breast cancer, demonstrating partial organ specificity (Supplementary Figures S10a and b; The Human Protein Atlas.org. https://www.proteinatlas.org/ENSG00000160862-AZGP1; accessed on November 07, 2022) [3]. We only verified a part of the ZAG function for the immune system. However, if the function of ZAG on the immune system is further established, ZAG may be a promising therapeutic target. Because breast cancer-derived ZAG can be detected in the serum [16], an assessment of serum ZAG may provide a minimally invasive marker reflecting the host immune response in the breast cancer microenvironment. Thus, our results may facilitate research with more clinical aspect in the future, and we too are conducting further analyses.

This study had some limitations, namely the relatively small number of patients enrolled in the in-house FCM dataset, the selection bias that may have affected the clinicopathological factors of the enrolled cases, and no subgroup analyses by the tumor subtype because of the small sample size [9, 23]. ZAG expression in breast cancer tissues was associated with decreased CD86 expression in Mφ (Fig. S6d); however, this association was not replicated in in vitro experiments (Fig. 4b, h, and n; Supplementary Figures S8b, h, and n). Besides, ZAG did not necessarily demonstrate a similar effect in M1/M2 and non-polarization models (Fig. 4). For instance, ZAG supplementation decreased CD80 expression in the M1 polarization model but increased it in the non-polarization model (Fig. 4a, m). Similarly, the effect of ZAG on PBMC-derived Mφ was not necessarily reproducible on THP-1 (Supplementary Figure S8). Supplementation of ZAG resulted in a statistically significant change in the expression of surface markers (Fig. 4); however, the change was so small that further investigation is required to determine whether it is clinically significant. The M1/M2 terminology has been introduced where M1s are pro-inflammatory and M2s are anti-inflammatory [47]. These states appear as two extremes with a large spectrum of macrophages in between [42]. Besides, some of these molecules (i.e., CD80, CD86, and MRC1) are bifunctional regarding their inflammatory or anti-inflammatory properties. Thus, further studies are required to determine the immunological role of ZAG in breast cancer. Lastly, as a future prospect of our study, validation using a mouse model is expected to provide further insights into the findings obtained in this study.

## Conclusions

In summary, our findings strongly suggest ZAG is a novel mediator of AR-dependent immunomodulation in the breast cancer microenvironment. *AZGP1*/ZAG was associated with an immunosuppressive phenotype and reduced infiltration of specific immune cell subsets, particularly Mφ, into breast cancer tissues. In the in vitro analysis, ZAG demonstrated some regulatory effects on the phenotypic change or Mφ function, suggesting its possible role as a regulator of tumor immune response in breast cancer microenvironment.

### Supplementary Information

Below is the link to the electronic supplementary material.

Supplementary file1 (PPTX 39585 kb)

## Data Availability

The datasets generated and/or analyzed during the current study shall be made available from the corresponding author upon reasonable request.

## Abbreviations

AR: Androgen receptor
CD: Cluster of Differentiation
CD16 + Mo: Non-classical monocytes
DCs: Dendritic cells
ER: Estrogen receptor
FCM: Flow cytometry
GM-CSF: Granulocyte macrophage colony-stimulating factor
GSEA: Gene Set Enrichment Analysis
HER2: Human epidermal growth factor receptor 2
HLA: Human leukocyte antigen
mDC: Myeloid DCs
MDSCs: Myeloid-derived suppressor cells
METABRIC: Molecular Taxonomy of Breast Cancer International Consortium
MFI: Mean fluorescence intensity
Mo/Mφ: Monocytes/macrophages
Mφ: Macrophage
NK: Natural killer cells
PBMC: Human peripheral blood mononuclear cells
PD-L1: Programmed death-ligand 1
PgR: Progesterone receptor
PMA: 10 Ng/ml Phorbol-12-myristate-13-acetate
SCAN-B: Sweden Cancerome Analysis Network-Breast
TIL: Tumor-infiltrating lymphocytes
ZAG: Alpha-2-glycoprotein 1, zinc-binding
