# Supplementary material for: Investigating the immunological function of alpha-2-glycoprotein 1, zinc-binding in regulating tumor response in the breast cancer microenvironment
Source: Cancer Immunol Immunother. 2024 Feb 13;73(3):42. doi: 10.1007/s00262-024-03629-1 (PMC10864576; doi:10.1007/s00262-024-03629-1)
Supplement: Supplementary file 1 — Supplementary file1 (PPTX 39585 kb) [file 262_2024_3629_MOESM1_ESM.pptx]

## Slide 1
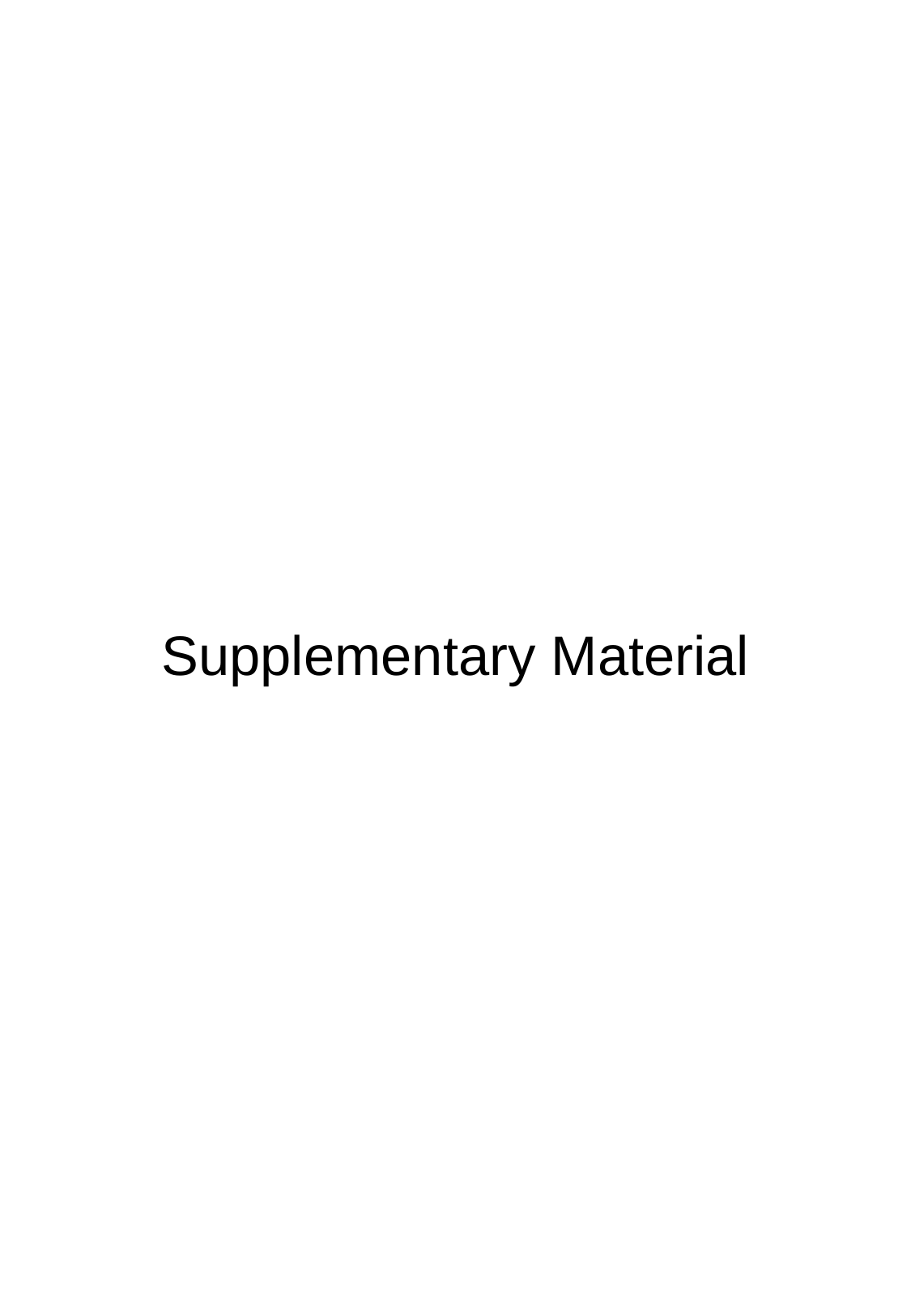

# Supplementary Material

## Slide 2
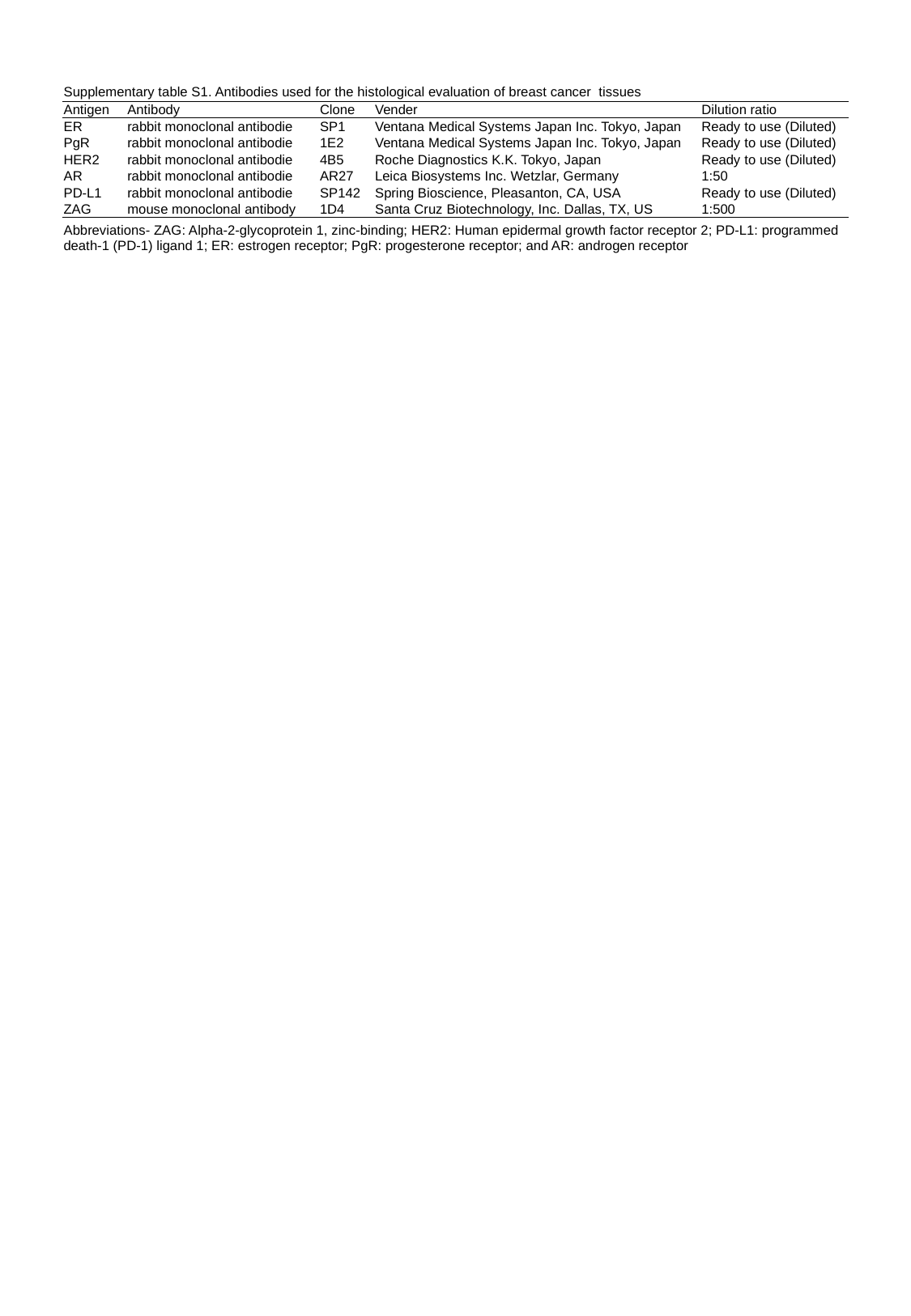

| Supplementary table S1. Antibodies used for the histological evaluation of breast cancer tissues | | | | |
| --- | --- | --- | --- | --- |
| Antigen | Antibody | Clone | Vender | Dilution ratio |
| ER | rabbit monoclonal antibodie | SP1 | Ventana Medical Systems Japan Inc. Tokyo, Japan | Ready to use (Diluted) |
| PgR | rabbit monoclonal antibodie | 1E2 | Ventana Medical Systems Japan Inc. Tokyo, Japan | Ready to use (Diluted) |
| HER2 | rabbit monoclonal antibodie | 4B5 | Roche Diagnostics K.K. Tokyo, Japan | Ready to use (Diluted) |
| AR | rabbit monoclonal antibodie | AR27 | Leica Biosystems Inc. Wetzlar, Germany | 1:50 |
| PD-L1 | rabbit monoclonal antibodie | SP142 | Spring Bioscience, Pleasanton, CA, USA | Ready to use (Diluted) |
| ZAG | mouse monoclonal antibody | 1D4 | Santa Cruz Biotechnology, Inc. Dallas, TX, US | 1:500 |
| Abbreviations- ZAG: Alpha-2-glycoprotein 1, zinc-binding; HER2: Human epidermal growth factor receptor 2; PD-L1: programmed death-1 (PD-1) ligand 1; ER: estrogen receptor; PgR: progesterone receptor; and AR: androgen receptor | | | | |

## Slide 3
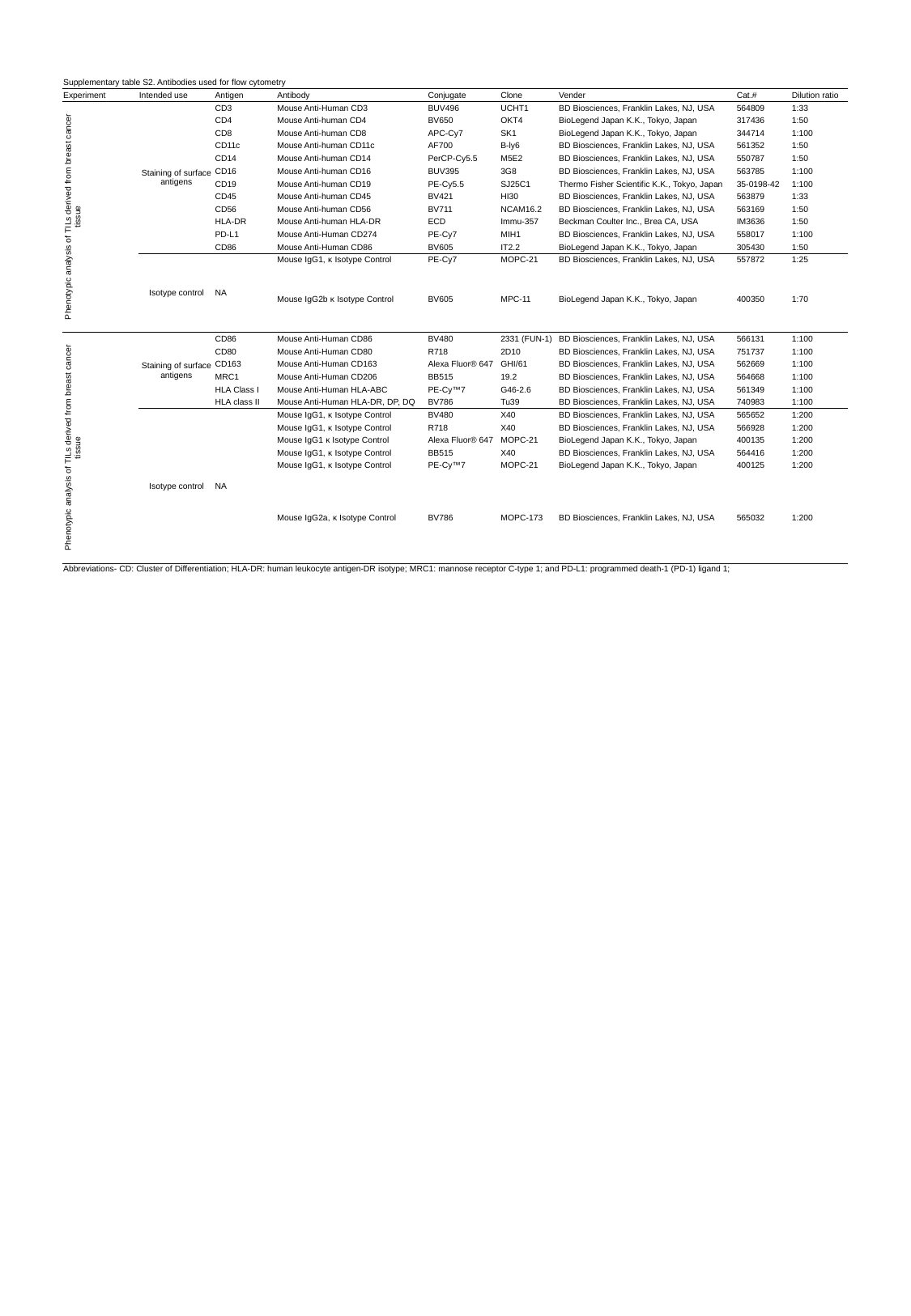

| Supplementary table S2. Antibodies used for flow cytometry | | | | | | | | |
| --- | --- | --- | --- | --- | --- | --- | --- | --- |
| Experiment | Intended use | Antigen | Antibody | Conjugate | Clone | Vender | Cat.# | Dilution ratio |
| Phenotypic analysis of TILs derived from breast cancer tissue | Staining of surface antigens | CD3 | Mouse Anti-Human CD3 | BUV496 | UCHT1 | BD Biosciences, Franklin Lakes, NJ, USA | 564809 | 1:33 |
| | | CD4 | Mouse Anti-human CD4 | BV650 | OKT4 | BioLegend Japan K.K., Tokyo, Japan | 317436 | 1:50 |
| | | CD8 | Mouse Anti-human CD8 | APC-Cy7 | SK1 | BioLegend Japan K.K., Tokyo, Japan | 344714 | 1:100 |
| | | CD11c | Mouse Anti-human CD11c | AF700 | B-ly6 | BD Biosciences, Franklin Lakes, NJ, USA | 561352 | 1:50 |
| | | CD14 | Mouse Anti-human CD14 | PerCP-Cy5.5 | M5E2 | BD Biosciences, Franklin Lakes, NJ, USA | 550787 | 1:50 |
| | | CD16 | Mouse Anti-human CD16 | BUV395 | 3G8 | BD Biosciences, Franklin Lakes, NJ, USA | 563785 | 1:100 |
| | | CD19 | Mouse Anti-human CD19 | PE-Cy5.5 | SJ25C1 | Thermo Fisher Scientific K.K., Tokyo, Japan | 35-0198-42 | 1:100 |
| | | CD45 | Mouse Anti-human CD45 | BV421 | HI30 | BD Biosciences, Franklin Lakes, NJ, USA | 563879 | 1:33 |
| | | CD56 | Mouse Anti-human CD56 | BV711 | NCAM16.2 | BD Biosciences, Franklin Lakes, NJ, USA | 563169 | 1:50 |
| | | HLA-DR | Mouse Anti-human HLA-DR | ECD | Immu-357 | Beckman Coulter Inc., Brea CA, USA | IM3636 | 1:50 |
| | | PD-L1 | Mouse Anti-Human CD274 | PE-Cy7 | MIH1 | BD Biosciences, Franklin Lakes, NJ, USA | 558017 | 1:100 |
| | | CD86 | Mouse Anti-Human CD86 | BV605 | IT2.2 | BioLegend Japan K.K., Tokyo, Japan | 305430 | 1:50 |
| | Isotype control | NA | Mouse IgG1, κ Isotype Control | PE-Cy7 | MOPC-21 | BD Biosciences, Franklin Lakes, NJ, USA | 557872 | 1:25 |
| | | | Mouse IgG2b κ Isotype Control | BV605 | MPC-11 | BioLegend Japan K.K., Tokyo, Japan | 400350 | 1:70 |
| Phenotypic analysis of TILs derived from breast cancer tissue | Staining of surface antigens | CD86 | Mouse Anti-Human CD86 | BV480 | 2331 (FUN-1) | BD Biosciences, Franklin Lakes, NJ, USA | 566131 | 1:100 |
| | | CD80 | Mouse Anti-Human CD80 | R718 | 2D10 | BD Biosciences, Franklin Lakes, NJ, USA | 751737 | 1:100 |
| | | CD163 | Mouse Anti-Human CD163 | Alexa Fluor® 647 | GHI/61 | BD Biosciences, Franklin Lakes, NJ, USA | 562669 | 1:100 |
| | | MRC1 | Mouse Anti-Human CD206 | BB515 | 19.2 | BD Biosciences, Franklin Lakes, NJ, USA | 564668 | 1:100 |
| | | HLA Class I | Mouse Anti-Human HLA-ABC | PE-Cy™7 | G46-2.6 | BD Biosciences, Franklin Lakes, NJ, USA | 561349 | 1:100 |
| | | HLA class II | Mouse Anti-Human HLA-DR, DP, DQ | BV786 | Tu39 | BD Biosciences, Franklin Lakes, NJ, USA | 740983 | 1:100 |
| | Isotype control | NA | Mouse IgG1, κ Isotype Control | BV480 | X40 | BD Biosciences, Franklin Lakes, NJ, USA | 565652 | 1:200 |
| | | | Mouse IgG1, κ Isotype Control | R718 | X40 | BD Biosciences, Franklin Lakes, NJ, USA | 566928 | 1:200 |
| | | | Mouse IgG1 κ Isotype Control | Alexa Fluor® 647 | MOPC-21 | BioLegend Japan K.K., Tokyo, Japan | 400135 | 1:200 |
| | | | Mouse IgG1, κ Isotype Control | BB515 | X40 | BD Biosciences, Franklin Lakes, NJ, USA | 564416 | 1:200 |
| | | | Mouse IgG1, κ Isotype Control | PE-Cy™7 | MOPC-21 | BioLegend Japan K.K., Tokyo, Japan | 400125 | 1:200 |
| | | | Mouse IgG2a, κ Isotype Control | BV786 | MOPC-173 | BD Biosciences, Franklin Lakes, NJ, USA | 565032 | 1:200 |
| Abbreviations- CD: Cluster of Differentiation; HLA-DR: human leukocyte antigen-DR isotype; MRC1: mannose receptor C-type 1; and PD-L1: programmed death-1 (PD-1) ligand 1; | | | | | | | | |

## Slide 4
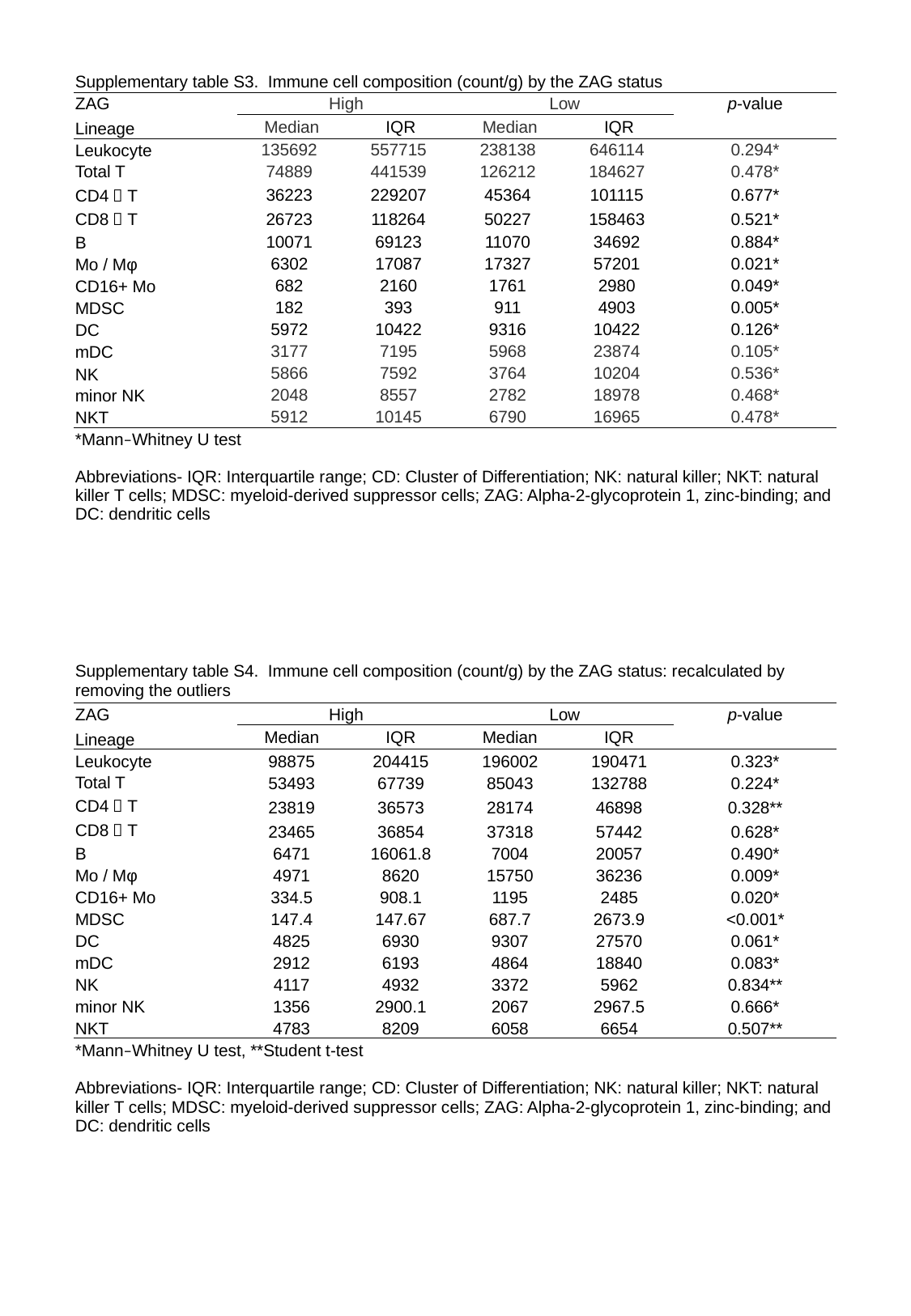

| Supplementary table S3. Immune cell composition (count/g) by the ZAG status | | | | | |
| --- | --- | --- | --- | --- | --- |
| ZAG | High | | Low | | p-value |
| Lineage | Median | IQR | Median | IQR | |
| Leukocyte | 135692 | 557715 | 238138 | 646114 | 0.294\* |
| Total T | 74889 | 441539 | 126212 | 184627 | 0.478\* |
| CD4＋T | 36223 | 229207 | 45364 | 101115 | 0.677\* |
| CD8＋T | 26723 | 118264 | 50227 | 158463 | 0.521\* |
| B | 10071 | 69123 | 11070 | 34692 | 0.884\* |
| Mo / Mφ | 6302 | 17087 | 17327 | 57201 | 0.021\* |
| CD16+ Mo | 682 | 2160 | 1761 | 2980 | 0.049\* |
| MDSC | 182 | 393 | 911 | 4903 | 0.005\* |
| DC | 5972 | 10422 | 9316 | 10422 | 0.126\* |
| mDC | 3177 | 7195 | 5968 | 23874 | 0.105\* |
| NK | 5866 | 7592 | 3764 | 10204 | 0.536\* |
| minor NK | 2048 | 8557 | 2782 | 18978 | 0.468\* |
| NKT | 5912 | 10145 | 6790 | 16965 | 0.478\* |
| \*Mann–Whitney U test | | | | | |
| Abbreviations- IQR: Interquartile range; CD: Cluster of Differentiation; NK: natural killer; NKT: natural killer T cells; MDSC: myeloid-derived suppressor cells; ZAG: Alpha-2-glycoprotein 1, zinc-binding; and DC: dendritic cells | | | | | |
| Supplementary table S4. Immune cell composition (count/g) by the ZAG status: recalculated by removing the outliers | | | | | |
| --- | --- | --- | --- | --- | --- |
| ZAG | High | | Low | | p-value |
| Lineage | Median | IQR | Median | IQR | |
| Leukocyte | 98875 | 204415 | 196002 | 190471 | 0.323\* |
| Total T | 53493 | 67739 | 85043 | 132788 | 0.224\* |
| CD4＋T | 23819 | 36573 | 28174 | 46898 | 0.328\*\* |
| CD8＋T | 23465 | 36854 | 37318 | 57442 | 0.628\* |
| B | 6471 | 16061.8 | 7004 | 20057 | 0.490\* |
| Mo / Mφ | 4971 | 8620 | 15750 | 36236 | 0.009\* |
| CD16+ Mo | 334.5 | 908.1 | 1195 | 2485 | 0.020\* |
| MDSC | 147.4 | 147.67 | 687.7 | 2673.9 | <0.001\* |
| DC | 4825 | 6930 | 9307 | 27570 | 0.061\* |
| mDC | 2912 | 6193 | 4864 | 18840 | 0.083\* |
| NK | 4117 | 4932 | 3372 | 5962 | 0.834\*\* |
| minor NK | 1356 | 2900.1 | 2067 | 2967.5 | 0.666\* |
| NKT | 4783 | 8209 | 6058 | 6654 | 0.507\*\* |
| \*Mann–Whitney U test, \*\*Student t-test | | | | | |
| Abbreviations- IQR: Interquartile range; CD: Cluster of Differentiation; NK: natural killer; NKT: natural killer T cells; MDSC: myeloid-derived suppressor cells; ZAG: Alpha-2-glycoprotein 1, zinc-binding; and DC: dendritic cells | | | | | |

## Slide 5
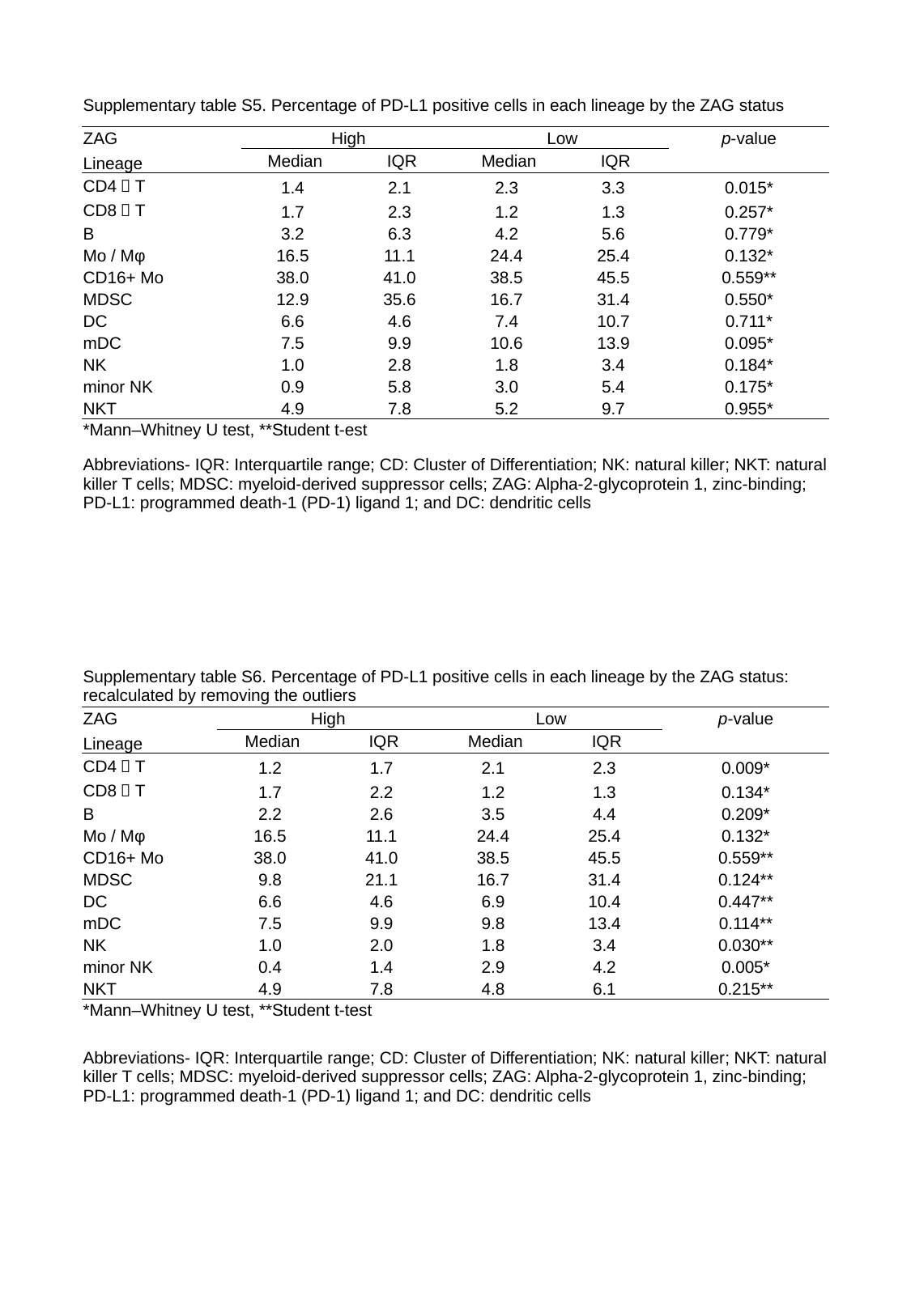

| Supplementary table S5. Percentage of PD-L1 positive cells in each lineage by the ZAG status | | | | | |
| --- | --- | --- | --- | --- | --- |
| ZAG | High | | Low | | p-value |
| Lineage | Median | IQR | Median | IQR | |
| CD4＋T | 1.4 | 2.1 | 2.3 | 3.3 | 0.015\* |
| CD8＋T | 1.7 | 2.3 | 1.2 | 1.3 | 0.257\* |
| B | 3.2 | 6.3 | 4.2 | 5.6 | 0.779\* |
| Mo / Mφ | 16.5 | 11.1 | 24.4 | 25.4 | 0.132\* |
| CD16+ Mo | 38.0 | 41.0 | 38.5 | 45.5 | 0.559\*\* |
| MDSC | 12.9 | 35.6 | 16.7 | 31.4 | 0.550\* |
| DC | 6.6 | 4.6 | 7.4 | 10.7 | 0.711\* |
| mDC | 7.5 | 9.9 | 10.6 | 13.9 | 0.095\* |
| NK | 1.0 | 2.8 | 1.8 | 3.4 | 0.184\* |
| minor NK | 0.9 | 5.8 | 3.0 | 5.4 | 0.175\* |
| NKT | 4.9 | 7.8 | 5.2 | 9.7 | 0.955\* |
| \*Mann–Whitney U test, \*\*Student t-est | | | | | |
| Abbreviations- IQR: Interquartile range; CD: Cluster of Differentiation; NK: natural killer; NKT: natural killer T cells; MDSC: myeloid-derived suppressor cells; ZAG: Alpha-2-glycoprotein 1, zinc-binding; PD-L1: programmed death-1 (PD-1) ligand 1; and DC: dendritic cells | | | | | |
| Supplementary table S6. Percentage of PD-L1 positive cells in each lineage by the ZAG status: recalculated by removing the outliers | | | | | |
| --- | --- | --- | --- | --- | --- |
| ZAG | High | | Low | | p-value |
| Lineage | Median | IQR | Median | IQR | |
| CD4＋T | 1.2 | 1.7 | 2.1 | 2.3 | 0.009\* |
| CD8＋T | 1.7 | 2.2 | 1.2 | 1.3 | 0.134\* |
| B | 2.2 | 2.6 | 3.5 | 4.4 | 0.209\* |
| Mo / Mφ | 16.5 | 11.1 | 24.4 | 25.4 | 0.132\* |
| CD16+ Mo | 38.0 | 41.0 | 38.5 | 45.5 | 0.559\*\* |
| MDSC | 9.8 | 21.1 | 16.7 | 31.4 | 0.124\*\* |
| DC | 6.6 | 4.6 | 6.9 | 10.4 | 0.447\*\* |
| mDC | 7.5 | 9.9 | 9.8 | 13.4 | 0.114\*\* |
| NK | 1.0 | 2.0 | 1.8 | 3.4 | 0.030\*\* |
| minor NK | 0.4 | 1.4 | 2.9 | 4.2 | 0.005\* |
| NKT | 4.9 | 7.8 | 4.8 | 6.1 | 0.215\*\* |
| \*Mann–Whitney U test, \*\*Student t-test | | | | | |
| Abbreviations- IQR: Interquartile range; CD: Cluster of Differentiation; NK: natural killer; NKT: natural killer T cells; MDSC: myeloid-derived suppressor cells; ZAG: Alpha-2-glycoprotein 1, zinc-binding; PD-L1: programmed death-1 (PD-1) ligand 1; and DC: dendritic cells | | | | | |

## Slide 6
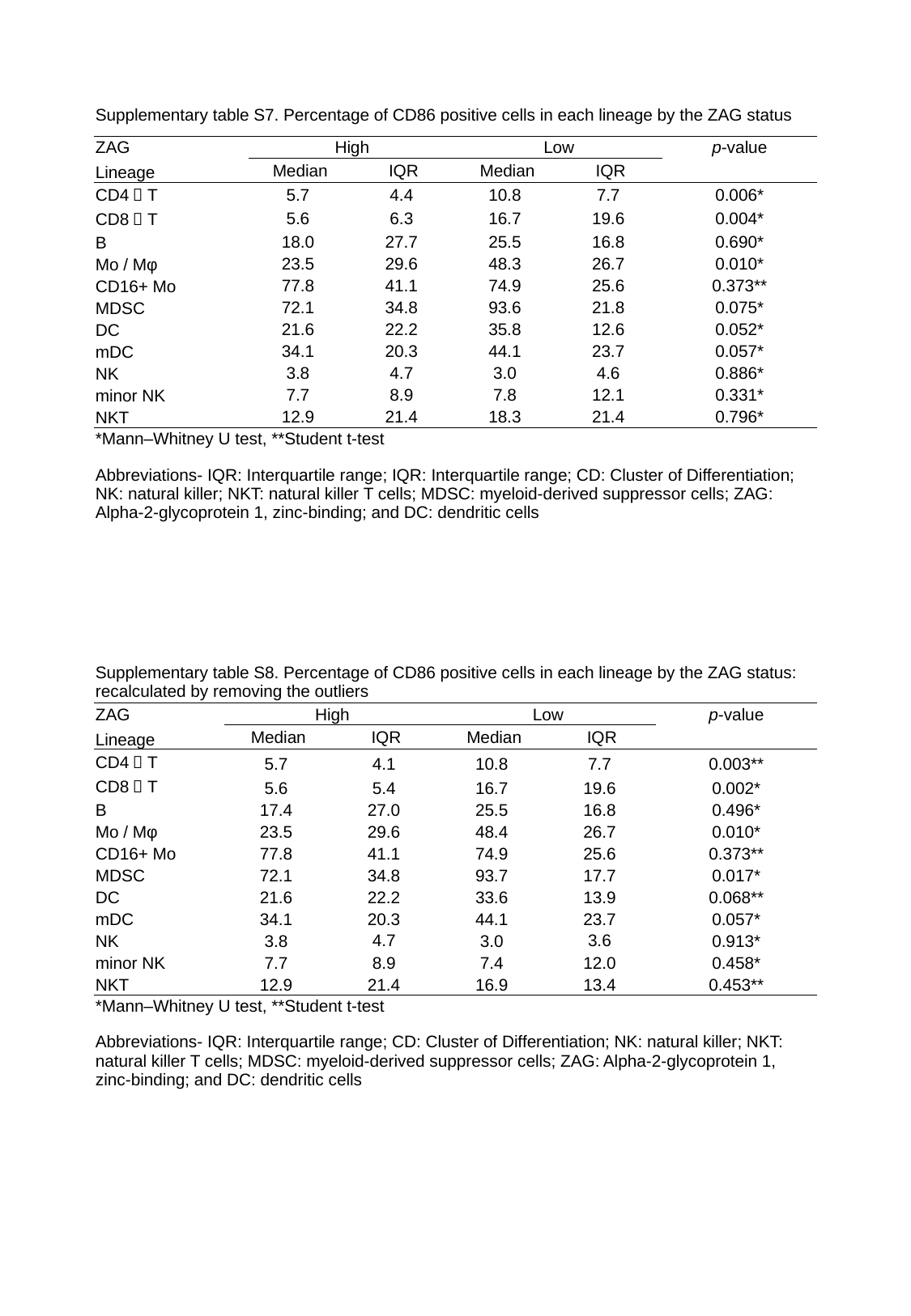

| Supplementary table S7. Percentage of CD86 positive cells in each lineage by the ZAG status | | | | | |
| --- | --- | --- | --- | --- | --- |
| ZAG | High | | Low | | p-value |
| Lineage | Median | IQR | Median | IQR | |
| CD4＋T | 5.7 | 4.4 | 10.8 | 7.7 | 0.006\* |
| CD8＋T | 5.6 | 6.3 | 16.7 | 19.6 | 0.004\* |
| B | 18.0 | 27.7 | 25.5 | 16.8 | 0.690\* |
| Mo / Mφ | 23.5 | 29.6 | 48.3 | 26.7 | 0.010\* |
| CD16+ Mo | 77.8 | 41.1 | 74.9 | 25.6 | 0.373\*\* |
| MDSC | 72.1 | 34.8 | 93.6 | 21.8 | 0.075\* |
| DC | 21.6 | 22.2 | 35.8 | 12.6 | 0.052\* |
| mDC | 34.1 | 20.3 | 44.1 | 23.7 | 0.057\* |
| NK | 3.8 | 4.7 | 3.0 | 4.6 | 0.886\* |
| minor NK | 7.7 | 8.9 | 7.8 | 12.1 | 0.331\* |
| NKT | 12.9 | 21.4 | 18.3 | 21.4 | 0.796\* |
| \*Mann–Whitney U test, \*\*Student t-test | | | | | |
| Abbreviations- IQR: Interquartile range; IQR: Interquartile range; CD: Cluster of Differentiation; NK: natural killer; NKT: natural killer T cells; MDSC: myeloid-derived suppressor cells; ZAG: Alpha-2-glycoprotein 1, zinc-binding; and DC: dendritic cells | | | | | |
| Supplementary table S8. Percentage of CD86 positive cells in each lineage by the ZAG status: recalculated by removing the outliers | | | | | |
| --- | --- | --- | --- | --- | --- |
| ZAG | High | | Low | | p-value |
| Lineage | Median | IQR | Median | IQR | |
| CD4＋T | 5.7 | 4.1 | 10.8 | 7.7 | 0.003\*\* |
| CD8＋T | 5.6 | 5.4 | 16.7 | 19.6 | 0.002\* |
| B | 17.4 | 27.0 | 25.5 | 16.8 | 0.496\* |
| Mo / Mφ | 23.5 | 29.6 | 48.4 | 26.7 | 0.010\* |
| CD16+ Mo | 77.8 | 41.1 | 74.9 | 25.6 | 0.373\*\* |
| MDSC | 72.1 | 34.8 | 93.7 | 17.7 | 0.017\* |
| DC | 21.6 | 22.2 | 33.6 | 13.9 | 0.068\*\* |
| mDC | 34.1 | 20.3 | 44.1 | 23.7 | 0.057\* |
| NK | 3.8 | 4.7 | 3.0 | 3.6 | 0.913\* |
| minor NK | 7.7 | 8.9 | 7.4 | 12.0 | 0.458\* |
| NKT | 12.9 | 21.4 | 16.9 | 13.4 | 0.453\*\* |
| \*Mann–Whitney U test, \*\*Student t-test | | | | | |
| Abbreviations- IQR: Interquartile range; CD: Cluster of Differentiation; NK: natural killer; NKT: natural killer T cells; MDSC: myeloid-derived suppressor cells; ZAG: Alpha-2-glycoprotein 1, zinc-binding; and DC: dendritic cells | | | | | |

## Slide 7
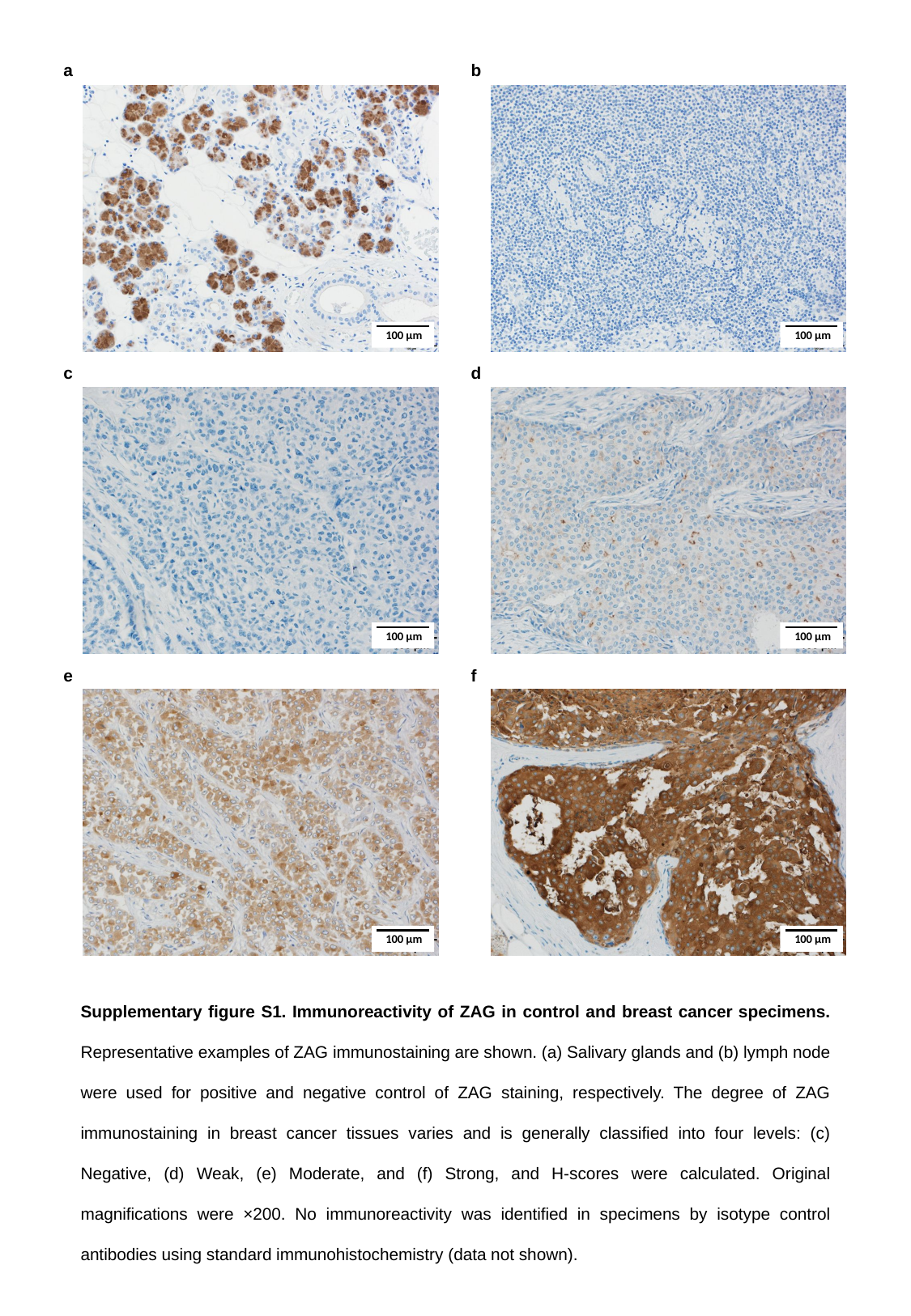

a
b
100 μm
100 μm
c
d
100 μm
100 μm
e
f
100 μm
100 μm
Supplementary figure S1. Immunoreactivity of ZAG in control and breast cancer specimens. Representative examples of ZAG immunostaining are shown. (a) Salivary glands and (b) lymph node were used for positive and negative control of ZAG staining, respectively. The degree of ZAG immunostaining in breast cancer tissues varies and is generally classified into four levels: (c) Negative, (d) Weak, (e) Moderate, and (f) Strong, and H-scores were calculated. Original magnifications were ×200. No immunoreactivity was identified in specimens by isotype control antibodies using standard immunohistochemistry (data not shown).

## Slide 8
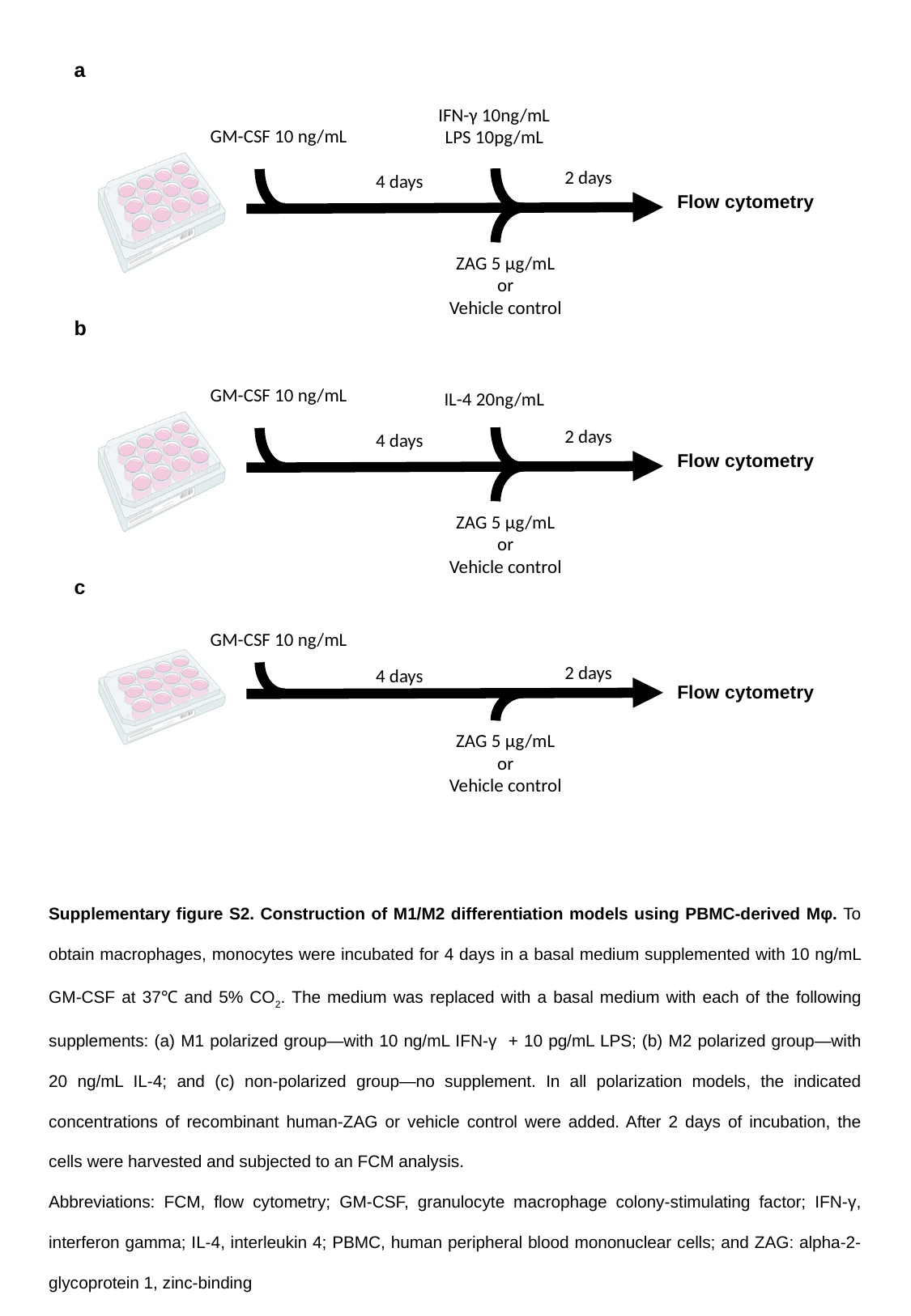

a
IFN-γ 10ng/mL
LPS 10pg/mL
GM-CSF 10 ng/mL
2 days
4 days
Flow cytometry
ZAG 5 μg/mL
or
Vehicle control
b
GM-CSF 10 ng/mL
IL-4 20ng/mL
2 days
4 days
Flow cytometry
ZAG 5 μg/mL
or
Vehicle control
c
GM-CSF 10 ng/mL
2 days
4 days
Flow cytometry
ZAG 5 μg/mL
or
Vehicle control
Supplementary figure S2. Construction of M1/M2 differentiation models using PBMC-derived Mφ. To obtain macrophages, monocytes were incubated for 4 days in a basal medium supplemented with 10 ng/mL GM-CSF at 37℃ and 5% CO2. The medium was replaced with a basal medium with each of the following supplements: (a) M1 polarized group—with 10 ng/mL IFN-γ + 10 pg/mL LPS; (b) M2 polarized group—with 20 ng/mL IL-4; and (c) non-polarized group—no supplement. In all polarization models, the indicated concentrations of recombinant human-ZAG or vehicle control were added. After 2 days of incubation, the cells were harvested and subjected to an FCM analysis.
Abbreviations: FCM, flow cytometry; GM-CSF, granulocyte macrophage colony-stimulating factor; IFN-γ, interferon gamma; IL-4, interleukin 4; PBMC, human peripheral blood mononuclear cells; and ZAG: alpha-2-glycoprotein 1, zinc-binding

## Slide 9
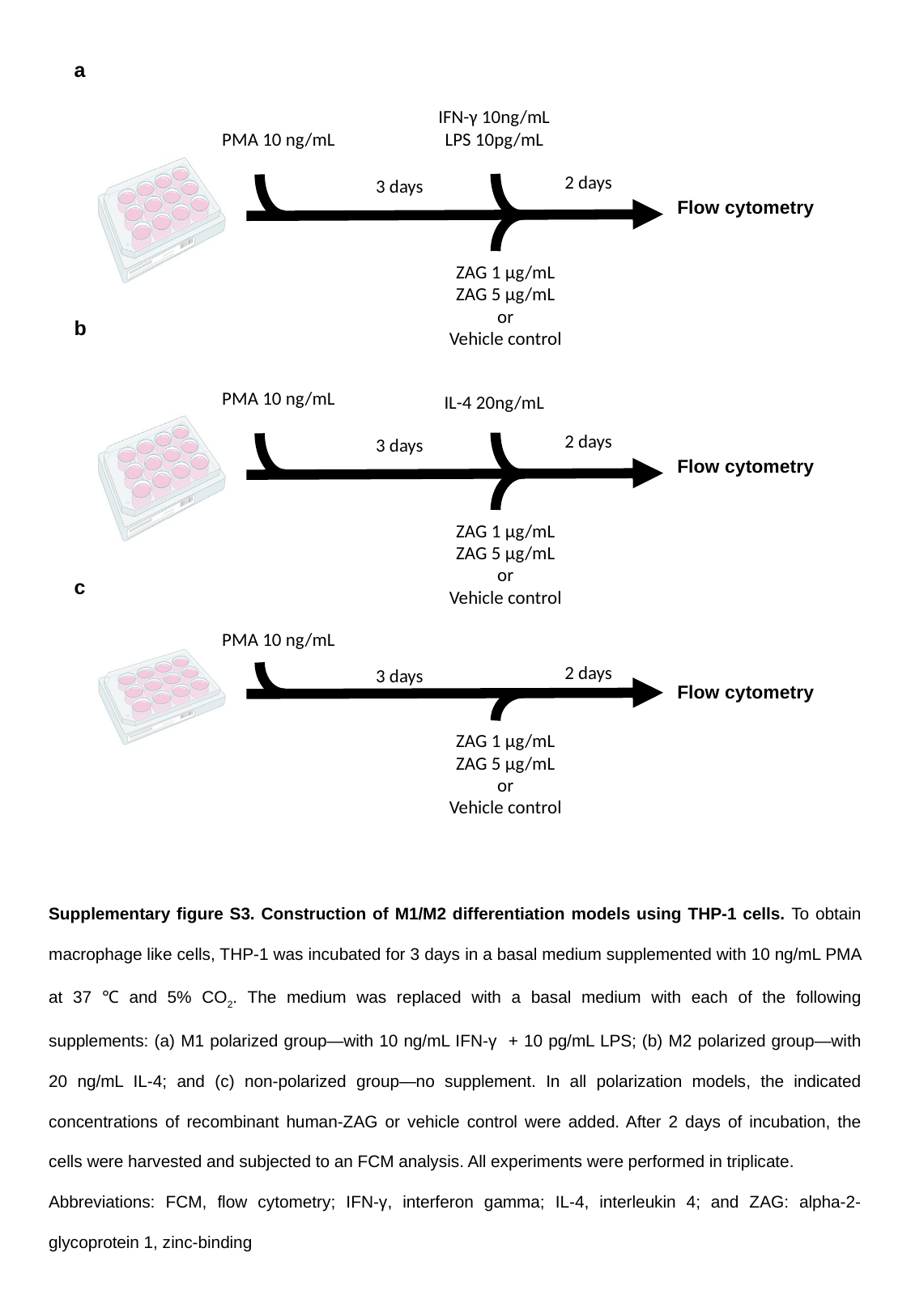

a
IFN-γ 10ng/mL
LPS 10pg/mL
PMA 10 ng/mL
2 days
3 days
Flow cytometry
ZAG 1 μg/mL
ZAG 5 μg/mL
or
Vehicle control
b
PMA 10 ng/mL
IL-4 20ng/mL
2 days
3 days
Flow cytometry
ZAG 1 μg/mL
ZAG 5 μg/mL
or
Vehicle control
c
PMA 10 ng/mL
2 days
3 days
Flow cytometry
ZAG 1 μg/mL
ZAG 5 μg/mL
or
Vehicle control
Supplementary figure S3. Construction of M1/M2 differentiation models using THP-1 cells. To obtain macrophage like cells, THP-1 was incubated for 3 days in a basal medium supplemented with 10 ng/mL PMA at 37 ℃ and 5% CO2. The medium was replaced with a basal medium with each of the following supplements: (a) M1 polarized group—with 10 ng/mL IFN-γ + 10 pg/mL LPS; (b) M2 polarized group—with 20 ng/mL IL-4; and (c) non-polarized group—no supplement. In all polarization models, the indicated concentrations of recombinant human-ZAG or vehicle control were added. After 2 days of incubation, the cells were harvested and subjected to an FCM analysis. All experiments were performed in triplicate.
Abbreviations: FCM, flow cytometry; IFN-γ, interferon gamma; IL-4, interleukin 4; and ZAG: alpha-2-glycoprotein 1, zinc-binding

## Slide 10
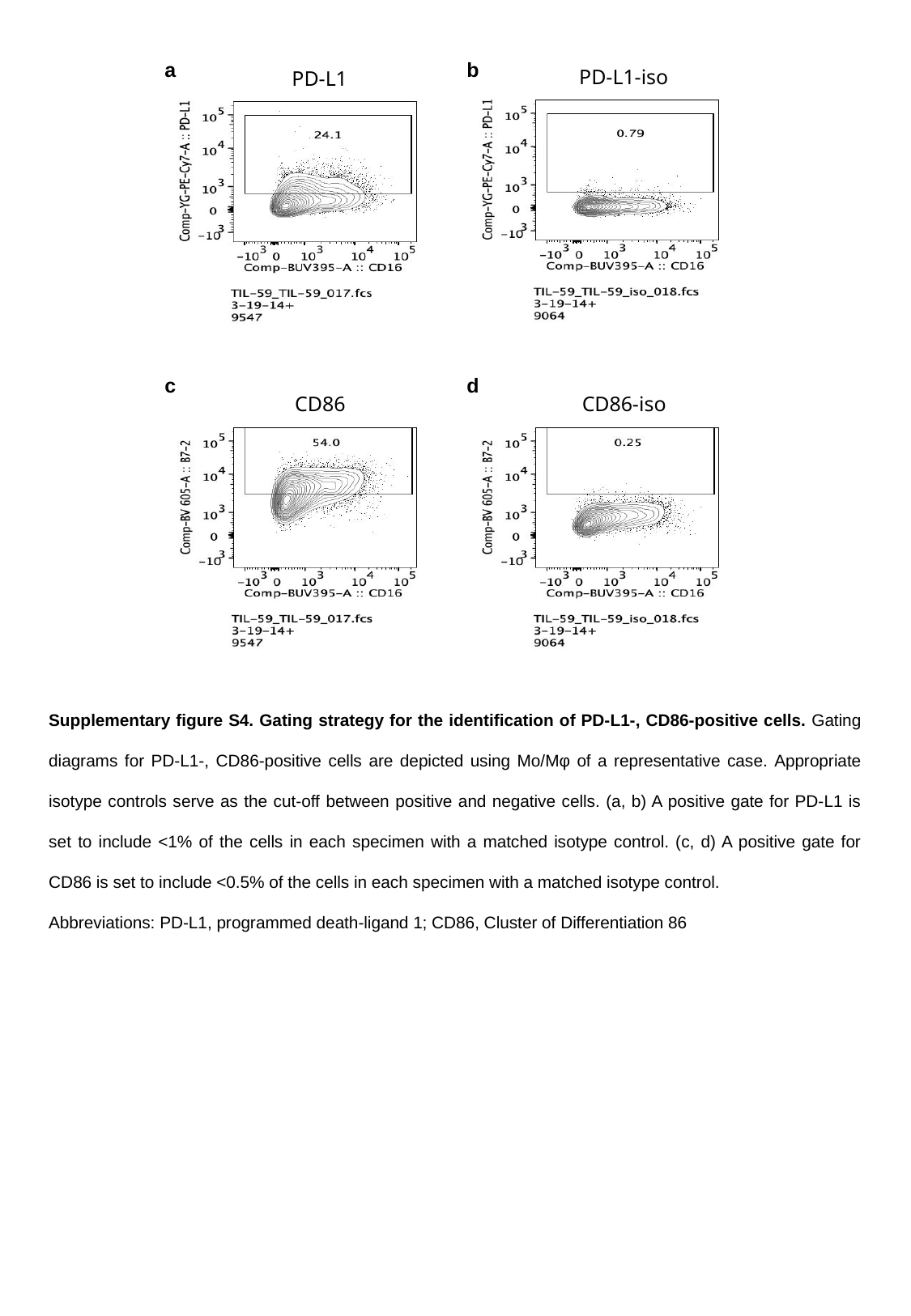

a
b
PD-L1-iso
PD-L1
c
d
CD86
CD86-iso
Supplementary figure S4. Gating strategy for the identification of PD-L1-, CD86-positive cells. Gating diagrams for PD-L1-, CD86-positive cells are depicted using Mo/Mφ of a representative case. Appropriate isotype controls serve as the cut-off between positive and negative cells. (a, b) A positive gate for PD-L1 is set to include <1% of the cells in each specimen with a matched isotype control. (c, d) A positive gate for CD86 is set to include <0.5% of the cells in each specimen with a matched isotype control.
Abbreviations: PD-L1, programmed death-ligand 1; CD86, Cluster of Differentiation 86

## Slide 11
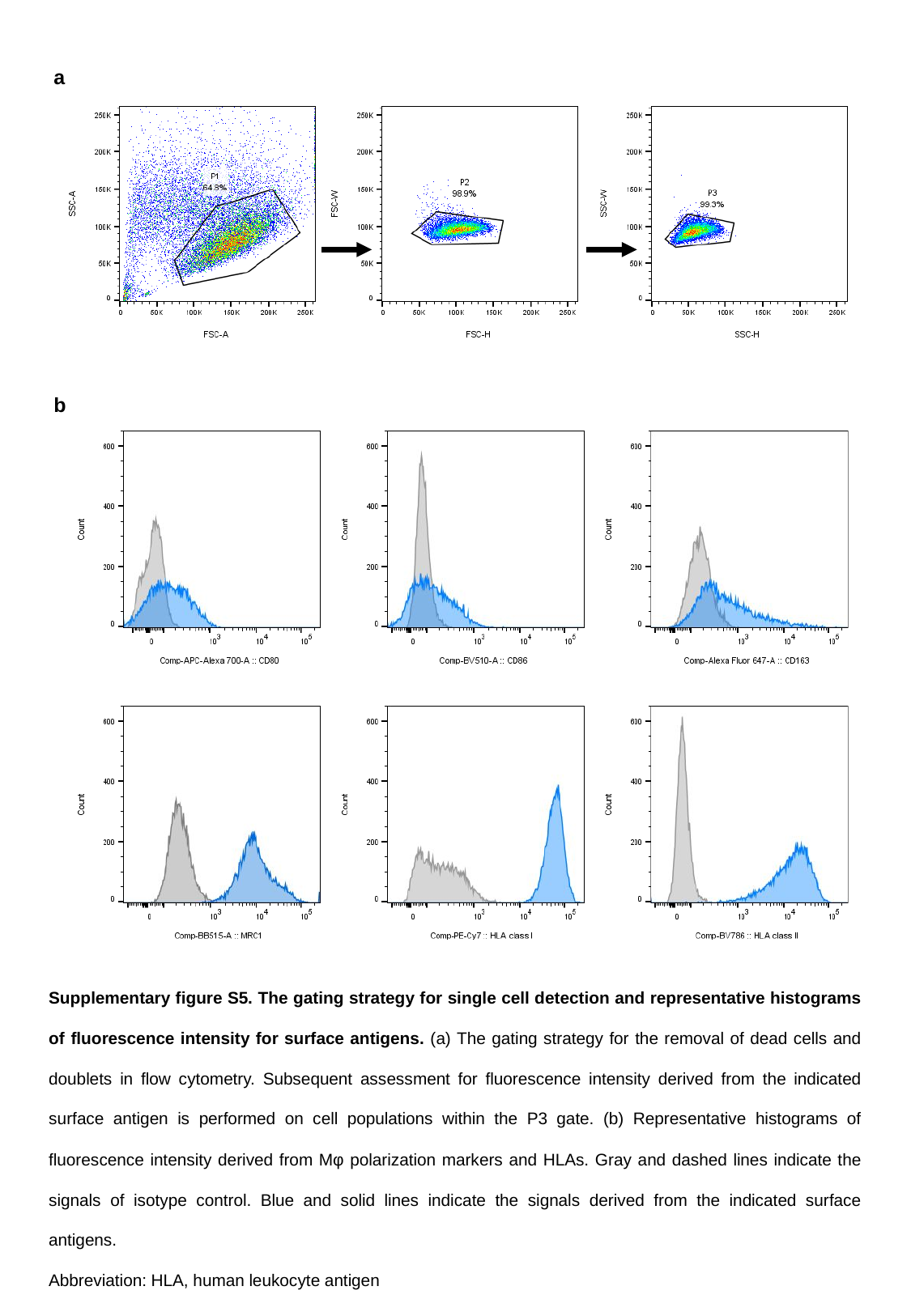

a
b
Supplementary figure S5. The gating strategy for single cell detection and representative histograms of fluorescence intensity for surface antigens. (a) The gating strategy for the removal of dead cells and doublets in flow cytometry. Subsequent assessment for fluorescence intensity derived from the indicated surface antigen is performed on cell populations within the P3 gate. (b) Representative histograms of fluorescence intensity derived from Mφ polarization markers and HLAs. Gray and dashed lines indicate the signals of isotype control. Blue and solid lines indicate the signals derived from the indicated surface antigens.
Abbreviation: HLA, human leukocyte antigen

## Slide 12
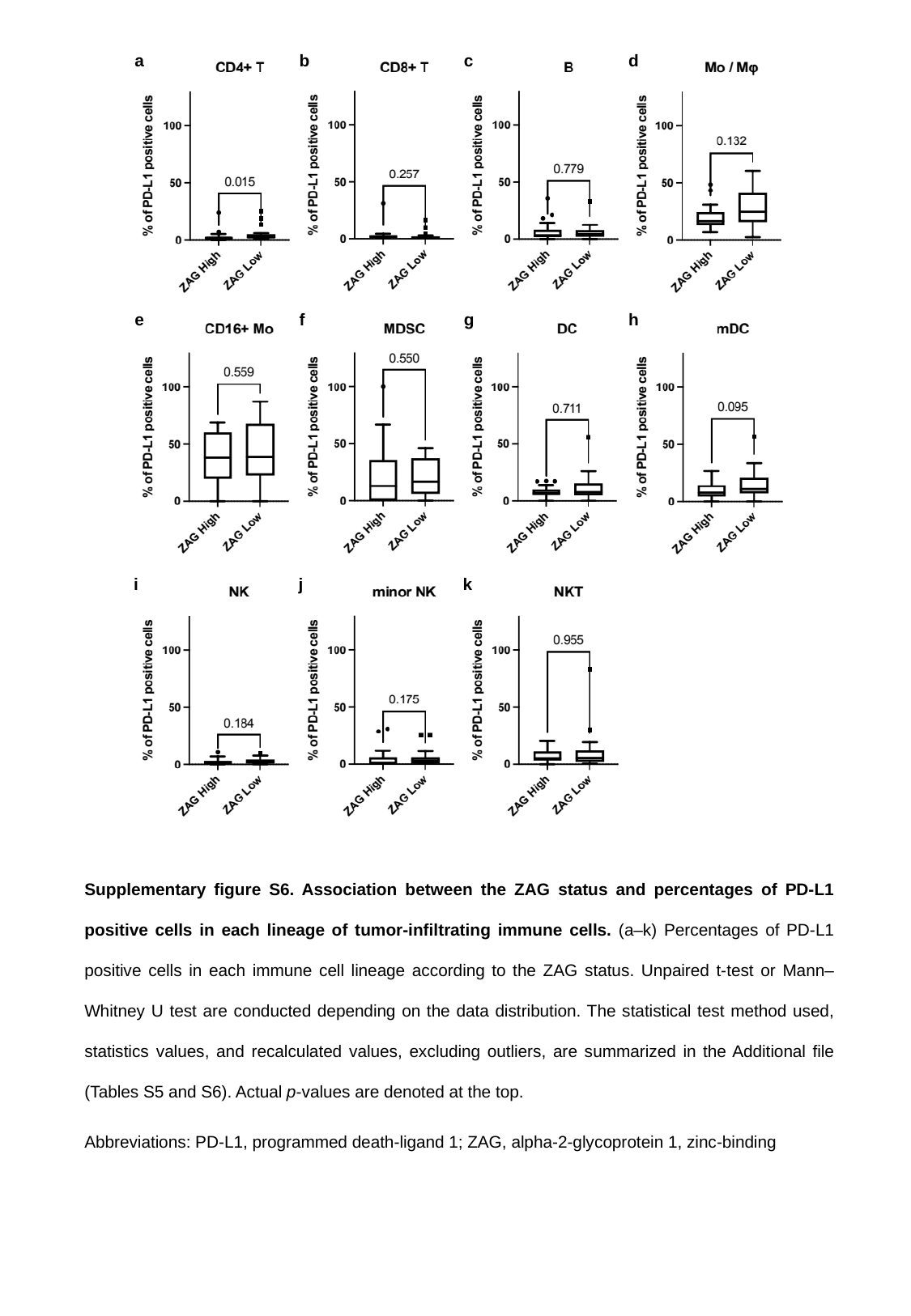

a
b
c
d
e
f
g
h
i
j
k
Supplementary figure S6. Association between the ZAG status and percentages of PD-L1 positive cells in each lineage of tumor-infiltrating immune cells. (a–k) Percentages of PD-L1 positive cells in each immune cell lineage according to the ZAG status. Unpaired t-test or Mann–Whitney U test are conducted depending on the data distribution. The statistical test method used, statistics values, and recalculated values, excluding outliers, are summarized in the Additional file (Tables S5 and S6). Actual p-values are denoted at the top.
Abbreviations: PD-L1, programmed death-ligand 1; ZAG, alpha-2-glycoprotein 1, zinc-binding

## Slide 13
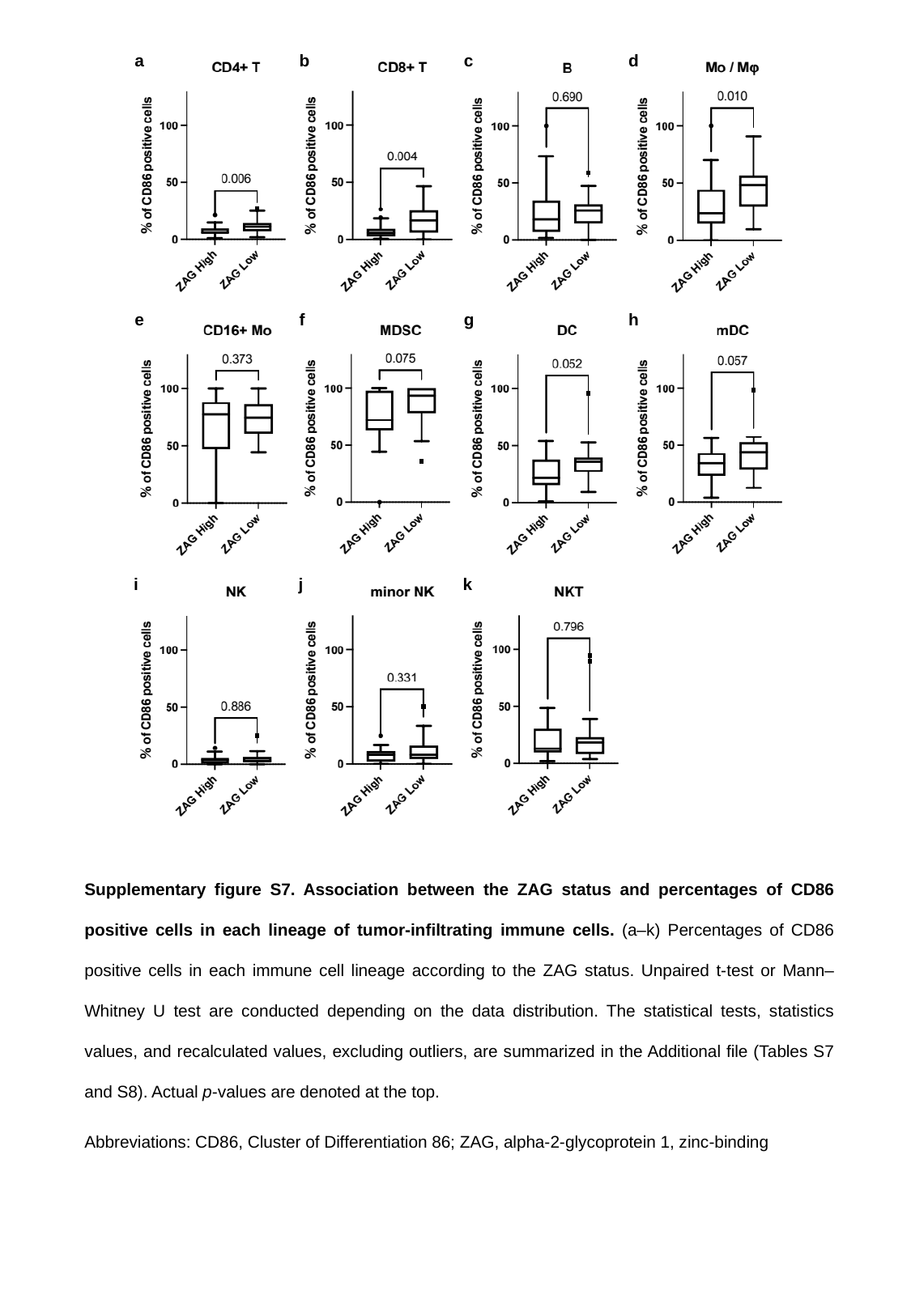

a
b
c
d
e
f
g
h
i
j
k
Supplementary figure S7. Association between the ZAG status and percentages of CD86 positive cells in each lineage of tumor-infiltrating immune cells. (a–k) Percentages of CD86 positive cells in each immune cell lineage according to the ZAG status. Unpaired t-test or Mann–Whitney U test are conducted depending on the data distribution. The statistical tests, statistics values, and recalculated values, excluding outliers, are summarized in the Additional file (Tables S7 and S8). Actual p-values are denoted at the top.
Abbreviations: CD86, Cluster of Differentiation 86; ZAG, alpha-2-glycoprotein 1, zinc-binding

## Slide 14
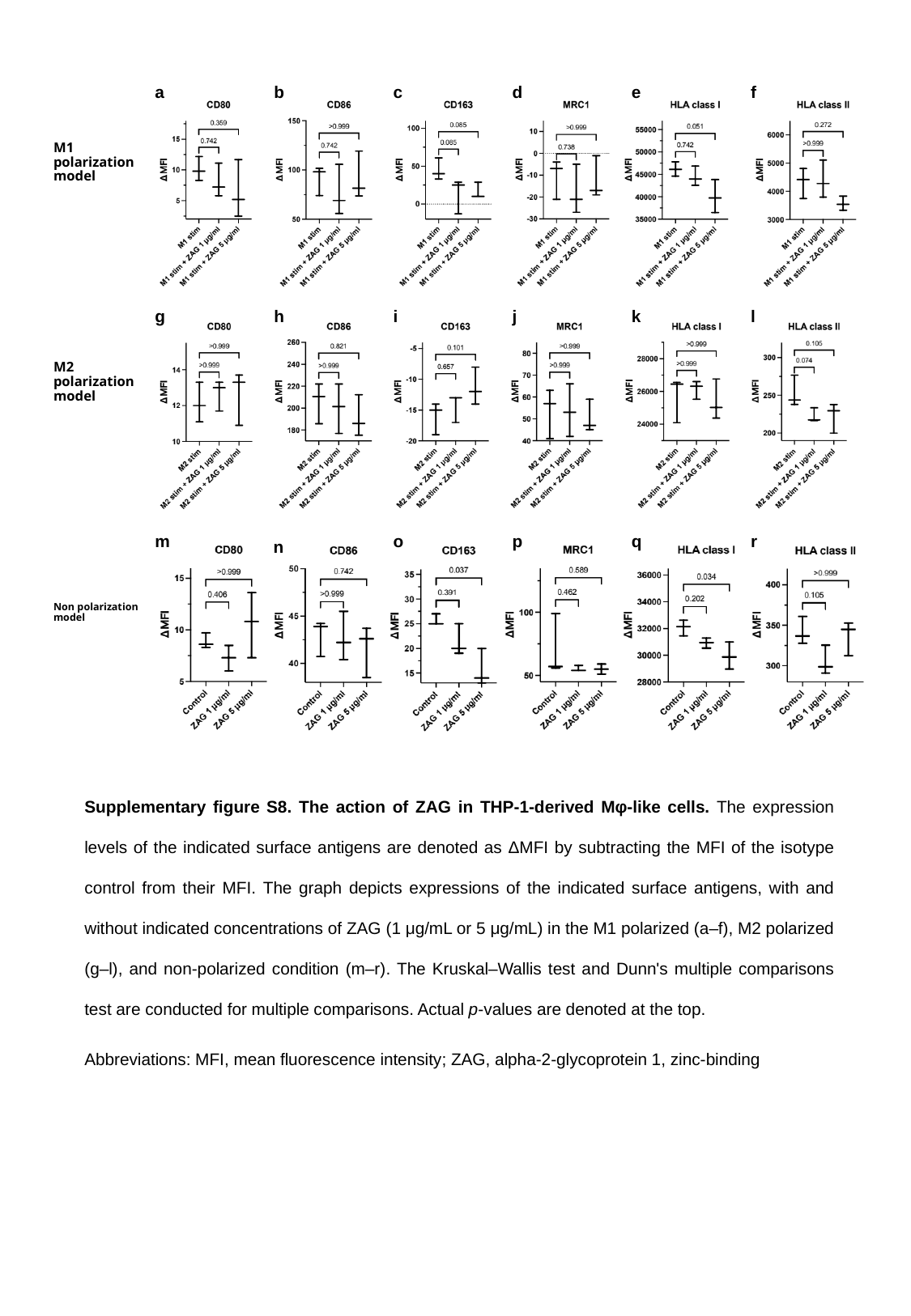

a
b
c
d
e
f
M1 polarization model
g
h
i
j
k
l
M2 polarization model
m
o
p
q
r
n
Non polarization model
Supplementary figure S8. The action of ZAG in THP-1-derived Mφ-like cells. The expression levels of the indicated surface antigens are denoted as ΔMFI by subtracting the MFI of the isotype control from their MFI. The graph depicts expressions of the indicated surface antigens, with and without indicated concentrations of ZAG (1 μg/mL or 5 μg/mL) in the M1 polarized (a–f), M2 polarized (g–l), and non-polarized condition (m–r). The Kruskal–Wallis test and Dunn's multiple comparisons test are conducted for multiple comparisons. Actual p-values are denoted at the top.
Abbreviations: MFI, mean fluorescence intensity; ZAG, alpha-2-glycoprotein 1, zinc-binding

## Slide 15
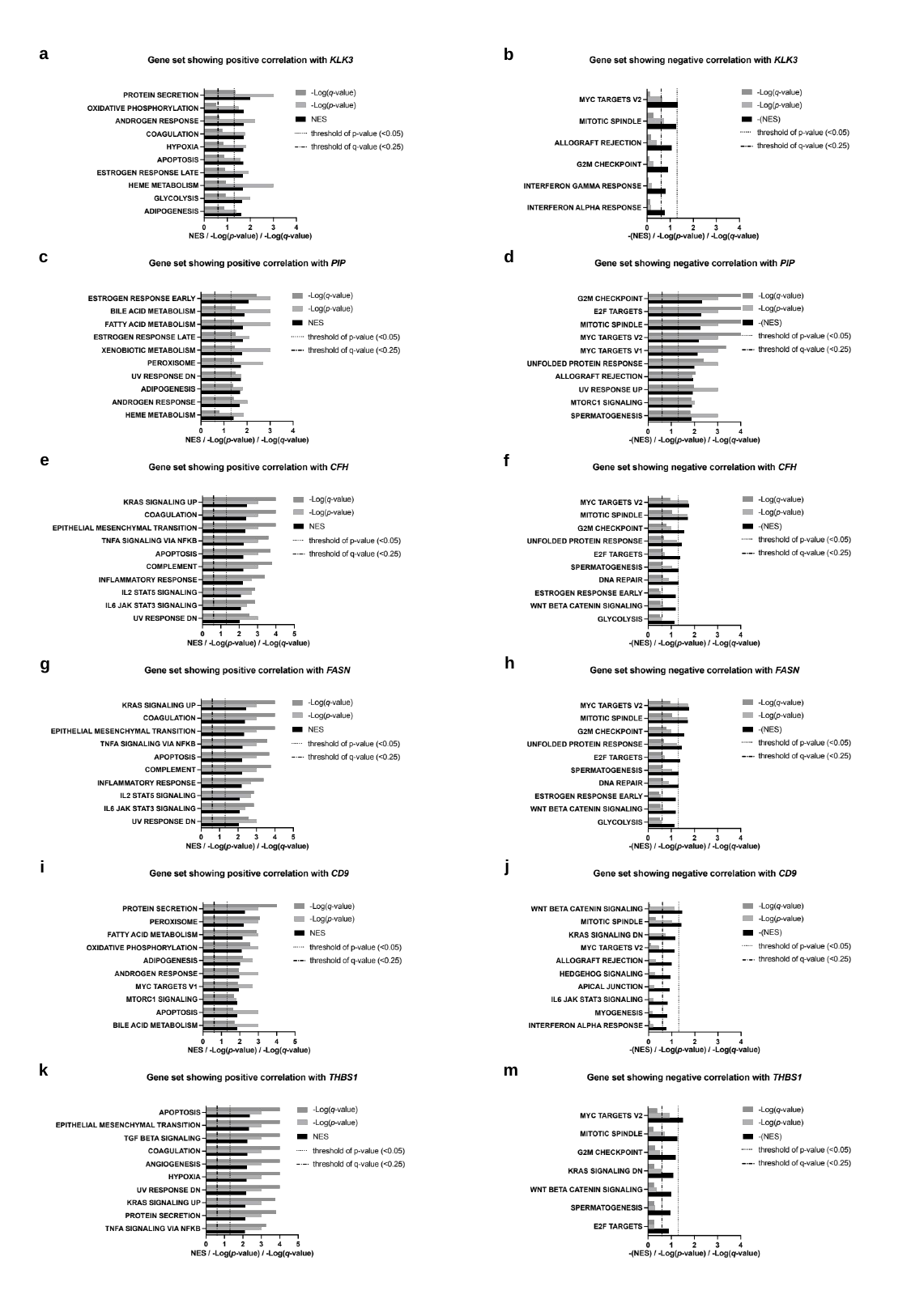

a
b
d
c
e
f
h
g
j
i
m
k

## Slide 16
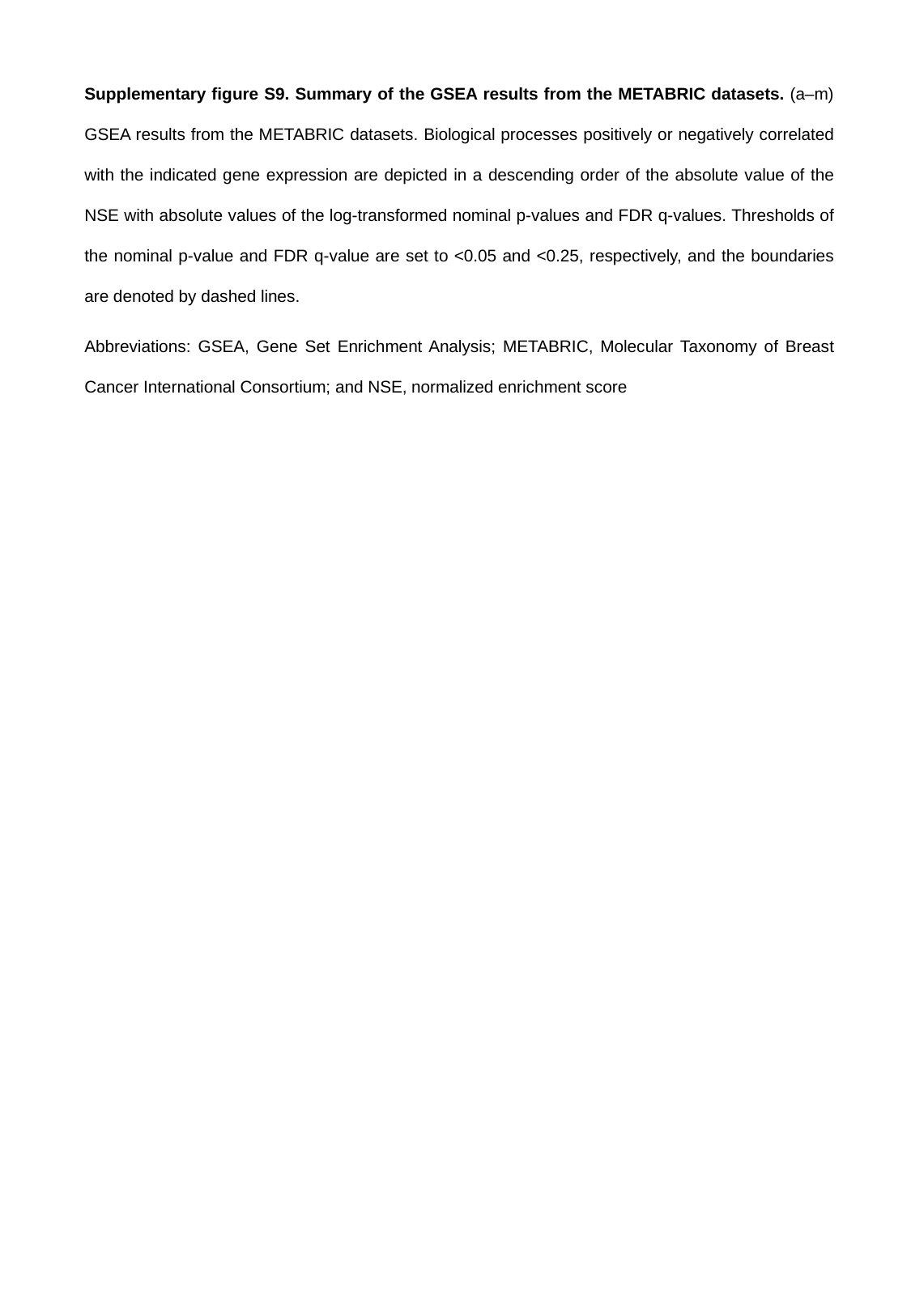

Supplementary figure S9. Summary of the GSEA results from the METABRIC datasets. (a–m) GSEA results from the METABRIC datasets. Biological processes positively or negatively correlated with the indicated gene expression are depicted in a descending order of the absolute value of the NSE with absolute values of the log-transformed nominal p-values and FDR q-values. Thresholds of the nominal p-value and FDR q-value are set to <0.05 and <0.25, respectively, and the boundaries are denoted by dashed lines.
Abbreviations: GSEA, Gene Set Enrichment Analysis; METABRIC, Molecular Taxonomy of Breast Cancer International Consortium; and NSE, normalized enrichment score

## Slide 17
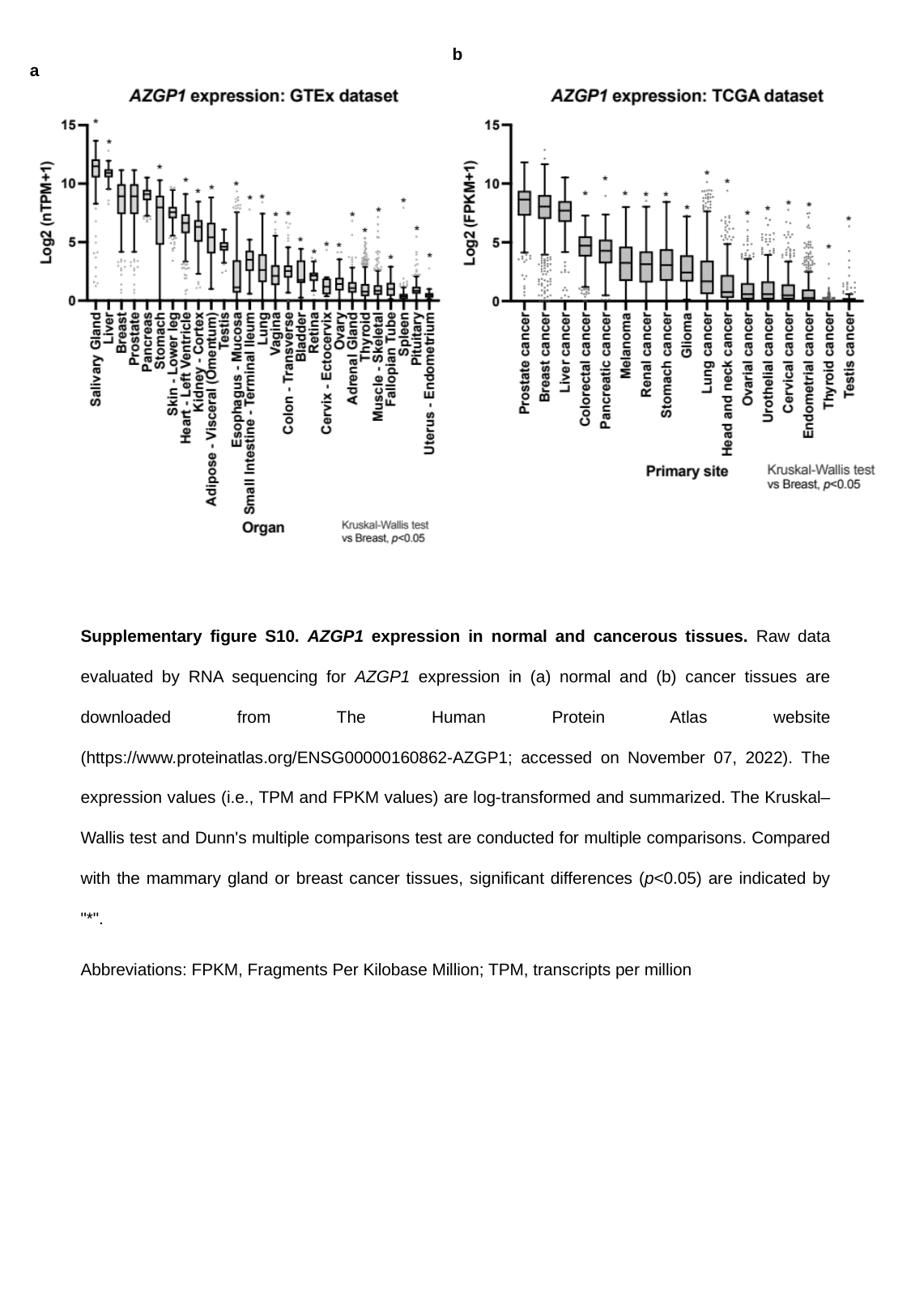

b
a
Supplementary figure S10. AZGP1 expression in normal and cancerous tissues. Raw data evaluated by RNA sequencing for AZGP1 expression in (a) normal and (b) cancer tissues are downloaded from The Human Protein Atlas website (https://www.proteinatlas.org/ENSG00000160862-AZGP1; accessed on November 07, 2022). The expression values (i.e., TPM and FPKM values) are log-transformed and summarized. The Kruskal–Wallis test and Dunn's multiple comparisons test are conducted for multiple comparisons. Compared with the mammary gland or breast cancer tissues, significant differences (p<0.05) are indicated by "*".
Abbreviations: FPKM, Fragments Per Kilobase Million; TPM, transcripts per million
